# Comparative Study of Mechanical Behavior and Failure Mechanisms in PA6- and PBT-Based Thermoplastic Fiber Metal Laminates

**DOI:** 10.3390/polym18121464

**Published:** 2026-06-11

**Authors:** Balcer Katarzyna, Boroński Dariusz, Skibicki Andrzej

**Affiliations:** Faculty of Mechanical Engineering, Bydgoszcz University of Science and Technology, Kaliskiego 7, 85-796 Bydgoszcz, Poland

**Keywords:** thermoplastic fiber metal laminate, thermoplastic composites, TFML, woven fabric composite, mechanical properties, SEM

## Abstract

Thermoplastic fiber metal laminates (TFMLs) are lightweight hybrid materials combining metallic layers with fiber-reinforced thermoplastic composites, offering a high strength-to-weight ratio. Existing studies indicate a limited range of polymer matrices used in such structures, most commonly polyamide 6 (PA6). In this work, polybutylene terephthalate (PBT) was selected as a potential alternative matrix because literature data indicate its lower moisture absorption and good dimensional stability compared with PA6. A comparative analysis of TFMLs based on aluminum and carbon fabric-reinforced composites with PA6 and PBT matrices was conducted. Static tensile tests were performed on base materials, composites, and laminates, supported by analytical modeling using the superposition method and fractographic analysis. The results showed that fiber orientation and polymer content significantly affect stiffness, strength, and damage evolution. Fiber orientation remains the governing factor, controlling load transfer and damage initiation. Laminates with 0/90° fibers exhibited the highest strength, while ±45° configurations showed reduced performance due to shear-dominated deformation. The polymer primarily acts as a matrix, ensuring structural integrity, with comparable mechanical properties for both systems. Delamination at the metal–composite interface was identified as the dominant failure mechanism.

## 1. Introduction

The development of modern industry places increasingly demanding requirements on structural materials, particularly in terms of strength, stiffness, and durability [1,2,3,4]. To date, both metals and fiber-reinforced composites have found widespread application. One important direction of development has been the design of fiber metal laminates (FMLs), which are hybrid materials combining the advantages of metals and fiber-reinforced composites [5]. In conventional structures of this type, however, epoxy resins belonging to the group of thermosetting polymers have been used predominantly [6,7,8,9,10,11]. These materials are characterized, among other things, by long manufacturing times and limited recyclability [12,13,14]. For this reason, increasing attention has been given to the use of thermoplastic polymers in fiber metal laminates, which, in addition to offering a favorable mechanical property-to-weight ratio, also enable shorter manufacturing times [15,16,17,18,19,20,21].

Thermoplastic fiber metal laminates (TFMLs) are currently considered a promising alternative to conventional fiber metal laminates with thermoset matrices [19]. Despite the growing interest in these structures, their use in real structural components still requires further investigation [22,23,24]. The systems reported in the literature are based mainly on polyamides, particularly PA6 and PA66 [19,25,26,27]. These materials exhibit good mechanical properties; however, their main limitation is their susceptibility to moisture absorption, which may affect the dimensional stability and long-term service performance of the laminate [26,28,29]. Therefore, the search for alternative thermoplastic matrices with more favorable functional properties appears justified. In this context, polybutylene terephthalate (PBT) is an interesting candidate material because literature data indicate its lower hygroscopicity and good dimensional stability compared with PA6 [30]. Despite these advantages, its application in TFML structures has not yet been widely described. PBT has been widely studied as a matrix in polymer composites, especially in systems reinforced with glass fibers [4,31,32,33,34,35]. However, these materials are less complex than TFMLs because they do not include metallic layers and metal–composite interfaces. One recent study reported aluminum/PBT laminates produced by hot pressing, but PBT was reinforced with short glass fibers in the form of a commercial granulate, and the analysis focused mainly on surface treatment and joint strength [36]. In contrast, PBT-based TFMLs with continuous woven carbon fabric have not yet been systematically studied, especially in terms of fabric orientation, polymer layer thickness, tensile behavior, and fracture morphology.

At the same time, it is well known that the mechanical properties and failure behavior of hybrid laminates depend on the reinforcement orientation, the proportion of constituent layers, and the quality of the metal–composite bond [37,38,39,40,41]. However, the analysis of mechanical properties alone does not fully explain the behavior of these materials, since similar stiffness and strength values may be accompanied by different mechanisms of damage initiation and propagation [42]. In this context, fracture surface analysis provides important information, as it makes it possible to assess the nature of failure and to identify phenomena such as delamination, interfacial debonding, matrix cracking, or fiber pull-out [43,44,45,46,47].

An additional challenge is the modeling of such materials, which usually requires a broad set of input data and extensive characterization of the base materials [48,49]. Advanced numerical approaches, including multi-scale finite element models, continuum damage mechanics, and cohesive zone modeling, are commonly used to analyze damage evolution, delamination, and impact response in laminated composite structures [50]. Therefore, alongside advanced methods, simpler analytical approaches are also important, such as the rule of mixtures-based superposition method, in which the laminate response is determined by summing the contributions of the individual layers under the assumption of strain compatibility [51].

In the present study, an original method for manufacturing thermoplastic fiber metal laminates composed of aluminum alloy layers and composite layers reinforced with carbon fabric was developed, using PA6 as the reference matrix material and PBT as the alternative matrix. For the structures produced in this way, a comparative analysis of their macroscopic mechanical properties, damage mechanisms, and fracture characteristics was carried out. The experimental program was supplemented by SEM fractographic analysis and superposition-based modeling, which made it possible to assess the agreement between the model predictions and the experimental results. Therefore, the comparison presented in this study should be understood as a comparison of PA6- and PBT-based TFML systems manufactured using bonding configurations suitable for each polymer, rather than as an isolated comparison of the matrix materials alone.

## 2. Materials and Fabrication Process

Thermoplastic composites were fabricated using polyamide PA6 (produced by POLITEM, Tekirdağ, Türkiye), polybutylene terephthalate PBT (produced by EPSAN, Bursa, Türkiye) and a twill-weave carbon fabric (supplied by Kordsa, İzmit, Türkiye). The material properties of the polymers are summarized in Table 1, those of the carbon fibers in Table 2, and the characteristics of the carbon fabric in Table 3.

The values listed in Table 1 are nominal supplier data for injection-molded materials and were used only for material identification. Experimentally determined density and tensile properties of the processed polymer plates are reported separately in Tables 7 and 10.

As metallic layers in the developed laminates, sheets of aluminum alloy 6061-T6 (Kaiser Aluminum Corporation, Franklin, TN, USA) were used, and their properties are summarized in Table 4. To enhance adhesion between the PA6-based composites and the aluminum alloy, a Pontacol 23.110 adhesive film with a thickness of 40 μm (PONTACOL AG, Schmitten, Switzerland) was applied. This film is based on modified polypropylene and is designed for durable bonding of metals and textiles. It has a density of 0.9 g/cm^3^, a melting temperature of 140–150 °C, and a recommended processing temperature range of 165–200 °C. The adhesive layer was placed between the PA6 (or composite) and the aluminum sheet. For the PBT-based TFMLs, a chemically etched FEP film (Holscot Advanced Polymers Ltd., Grantham, UK) with a thickness of 100 μm was used. The film, made from a fluorinated ethylene–propylene copolymer, exhibits excellent chemical resistance, thermal stability (–200 to >200 °C), and UV resistance. A surface layer of modified polypropylene was applied to the FEP film to ensure durable bonding between the PBT layer and the aluminum alloy 6061-T6 sheets.

**Figure 1:**
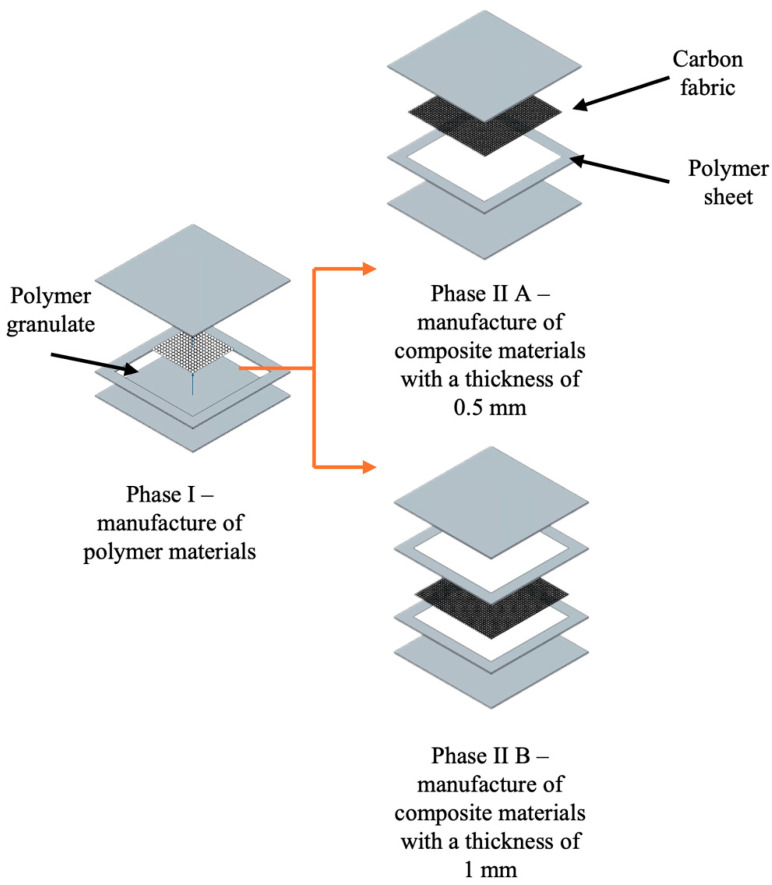
Schematic of the fabrication process of fabric-reinforced thermoplastic composites.

In the previous study [52], the preparation procedure and basic mechanical properties of 1 mm thick PA6- and PBT-based CFRTP composites were presented. These composites correspond to the lower fiber content group used in the present laminate analysis. In the present work, additional 0.5 mm thick CFRTP composites were manufactured and tested using a modified fabrication method with a single, thinner polymer layer. Both the 1 mm and 0.5 mm composites are included in this study because they were used as constituent layers in the analyzed TFMLs. In the first stage, a monolithic polymer plate with a thickness of 0.5 mm was formed within a steel frame. A single layer of carbon fabric was then placed on its surface, and the assembly was consolidated in a hydraulic press under controlled temperature and pressure conditions. As a result, a composite with an approximate thickness of 0.5 mm was obtained, consisting of one polymer layer and one layer of carbon fabric. A schematic representation of the fabrication process of the composite base materials is shown in Figure 1.

**Figure 2:**
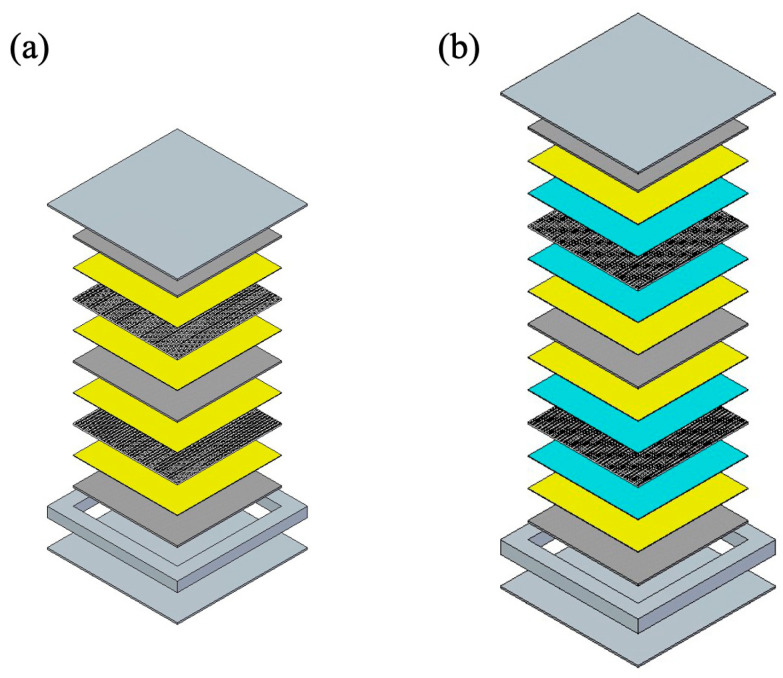
Layer configuration of the TFMLs: (**a**) with a PA6 matrix, (**b**) with a PBT matrix. The aluminum layers are marked in grey, the carbon fabric in black, the adhesive film 23.110 in yellow, and the FEP film in blue.

TFMLs were fabricated by hot pressing using the previously prepared thermoplastic composites. Prior to processing, the aluminum sheets were subjected to a mechanical–chemical surface treatment, which included sanding with 1000-grit abrasive paper, rinsing with demineralized water, degreasing with isopropanol, and drying. Two layers of polymer or prepreg (depending on the laminate configuration) were placed between the aluminum sheets. In the laminates based on PA6, an adhesive film 23.110 (PONTACOL AG, Schmitten, Switzerland) made of modified polypropylene was used, whereas in the PBT-based laminates, a chemically etched FEP film (ethylene-propylene copolymer) (Holscot Advanced Polymers Ltd, Grantham, UK) was applied, followed by the 23.110 adhesive layer (Figure 2). The modified bonding configuration used for the PBT-based laminates was introduced because preliminary trials with only the 23.110 adhesive film, as used for the PA6-based laminates, did not provide stable PBT-based laminates and resulted in layer separation after pressing. Therefore, the additional chemically etched FEP film was applied as a technological solution necessary to obtain stable PBT-based TFML structures. Consequently, the PA6- and PBT-based laminates should not be treated as systems with identical bonding conditions. The influence of the different adhesive configurations was not separated in the present study and is therefore considered a limitation.

Depending on the fiber volume fraction, which corresponded to the thickness of the polymer plate used in the composite, TFMLs with total thicknesses of 2.5 mm and 3.5 mm were fabricated. The processing parameters used during the hot pressing of the TFMLs are summarized in Table 5.

The pressing temperatures were selected based on the processing recommendations provided in the manufacturers’ datasheets for PA6 and PBT. No additional optimization of the pressing temperature was performed in this study. Therefore, possible effects of processing temperature on crystallinity, residual stresses, and the degree of consolidation should be considered a limitation of the present work.

Table 6 presents all the investigated materials along with their corresponding designations, including both the base composites and those incorporated into the TFMLs.

## 3. Methods

In the first stage, a static tensile test was performed on the polymer base materials and on the composites produced using these polymers. This test made it possible to determine the mechanical properties that serve as a reference point for the analysis of the mechanical response of the laminates. The obtained data were used for modeling, which may enable the prediction of the mechanical properties of laminates based on their constituent materials, without the need for extensive experimental testing. The laminates were also subjected to static tensile tests to verify the model in relation to the experimentally obtained stress–strain curves. The experimental program was complemented by a fractographic analysis of the fracture surfaces, which provided insight into the failure mechanisms and allowed the correlation of microscopic observations with the mechanical test results.

Static tensile tests were conducted for the following groups of materials: polymer base materials (PA6 and PBT), the base metallic material (aluminum alloy), thermoplastic composites reinforced with carbon fabric (CFRTP) with different fiber volume fractions [52], as well as adhesive films and TFMLs. The tensile tests for polymers (PA6, PBT), the aluminum alloy, and the composites with higher fiber content were carried out using an INSTRON 5966 (Instron, Norwood, MA, USA) universal testing machine, whereas the composites with lower fiber content and the TFMLs were tested using an INSTRON 8502 testing machine (Instron, Norwood, MA, USA). In both testing machines, the specimen elongation was recorded directly in the gauge section using the same type of static extensometer type 2630-110 (Instron, Norwood, MA, USA), with a gauge length of 50 mm and a measurement range of ±100%. The deformation field was additionally monitored using a DIC camera system. The static tensile tests of the adhesive films, used to bond the polymer and metal layers, were performed using a Zwick Roell Z030 (ZwickRoell GmbH & Co. KG, Ulm, Germany) testing machine.

The tests of the polymers and composites were carried out in accordance with the EN ISO 527-1 standard [53], while the tensile tests of the aluminum alloy were performed in accordance with EN ISO 6892-1 [54]. The thermoplastic composites and TFMLs were tested at a loading rate of 1 mm/min. The static tensile tests of the adhesive films were performed at a loading rate of 50 mm/min, following the requirements of EN ISO 527-3 [55]. The yield strength was determined using the 0.2% offset method. For materials for which abrupt brittle failure occurred without plastic deformation, σ_Y_ was not reported.

**Figure 3:**
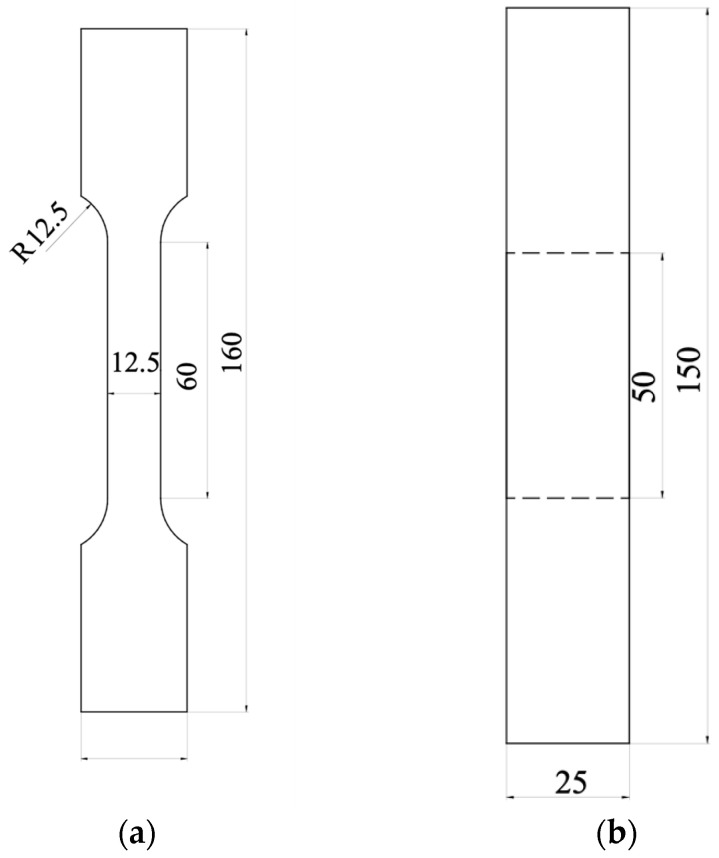
Test specimens: (**a**) static tensile test specimen (type A), (**b**) adhesive film tensile test specimen (type B).

For the static tensile tests, type A dog-bone specimens were used [56,57] (Figure 3a), while type B specimens prepared according to EN ISO 527-3 were used for the tensile tests of the adhesive films (Figure 3b).

The specimens for mechanical testing, made of polymer, composite, and laminate materials, were cut using a milling technique. Although water-jet cutting is recommended in the literature [19], preliminary trials showed that this method promoted delamination; therefore, milling was selected. Single-flute end mills with a diameter of 3 mm were used for specimen preparation. To prevent potential changes in the material properties during the milling process, the specimen temperature was monitored using a thermal imaging camera. The prepared specimens were subjected to a macroscopic evaluation of the cross-section to assess the distribution and uniformity of carbon fiber reinforcement, ensuring consistent fiber contribution to load transfer.

The density of the composites was determined according to the EN ISO 1183-1 [58] standard using the immersion method. The measurement was carried out with a balance equipped with a hydrostatic attachment AD50. The procedure involved determining the mass of each specimen in air and after immersion in distilled water with a density of 1000 kg/m^3^.

The morphology of the fracture surfaces of the woven composites and laminates subjected to static tensile testing, as well as the analysis of their failure mechanisms, was carried out using a JEOL JSM-6480LV scanning electron microscope (JEOL Ltd., Tokyo, Japan). The observations were performed in secondary electron imaging (SEI) mode at an accelerating voltage of 5 kV and at various magnifications.

**Figure 4:**
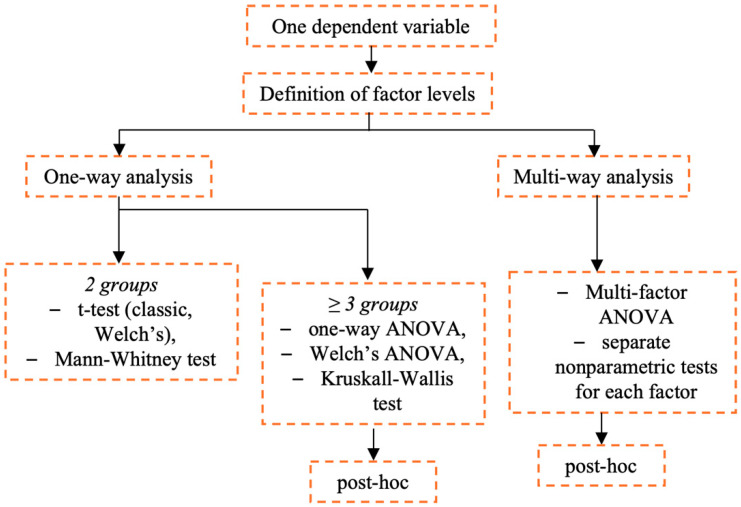
Decision framework for selecting statistical analysis methods.

For each material configuration, three specimens were tested (*n* = 3). The same number of specimens was used for the adhesive films. Due to the limited sample size, the statistical analysis should be treated as supportive and exploratory rather than as a fully powered statistical validation. A statistical analysis was carried out to assess the influence of the investigated factors on the mechanical properties of the examined materials. The analysis covered composites (polymer type and fiber orientation) and laminates (polymer content, polymer type, and fiber orientation or its absence). Accordingly, the selection of statistical tests followed the decision framework shown in Figure 4, developed on the basis of [59,60,61,62,63,64,65,66,67,68]. The analyses were carried out using IBM SPSS Statistics software (IBM Corp., Armonk, NY, USA).

The selection of parametric and nonparametric tests, along with their underlying assumptions, was based on the recommendations found in the literature [64,69]. Prior to choosing the appropriate statistical tests, the normality of the data distribution was verified using the Shapiro–Wilk or Kolmogorov–Smirnov test, and the homogeneity of variances was examined using Levene’s test. Post hoc analyses were performed only when the analyzed factor had more than two levels (e.g., fabric orientation: none, 0/90°, ±45°) and showed a statistically significant effect in the main test. Three main post hoc tests were applied: the Mann–Whitney test with Bonferroni correction (used after the Kruskal–Wallis test when the data did not follow a normal distribution), the Tamhane’s T2 post hoc test (applied after Welch’s ANOVA when the assumption of homogeneity of variances was violated), and the Tukey HSD post hoc test (performed following a one-way ANOVA).

## 4. Results and Discussion

### 4.1. Density

This section presents the results of measurements and the analysis of the density of base materials, composites, and laminates. Density is a key parameter that, in the case of composites, enables the calculation of the fiber volume fraction, and in the case of laminates, serves as the basis for determining mechanical parameters, including the specific modulus E_spec_ and specific strength σ_spec_, described in more detail in Section 4.2.

#### 4.1.1. Base Materials and Thermoplastic Composites

Table 7 presents a summary of the density measurements obtained for the base materials and the corresponding CFRTP composites fabricated from them. The experimentally measured density values listed in Table 7 were used for the calculation of the fiber volume fraction. Therefore, the fiber volume fraction was calculated using the measured density of the polymer matrix and composite, rather than the nominal supplier data presented in Table 1. Since the compared composites contained the same constituents, i.e., the same polymer and fiber contents, fiber orientation was not expected to affect density; therefore, in this part of the analysis, the composite designation was based on the polymer type and polymer content.

The analysis of the obtained density values made it possible to determine the fiber volume fraction f_f_ of the investigated composites. The rule of mixtures was applied to calculate the fiber volume content according to Equation (1) [70]. The calculated values of the fiber volume fraction are summarized in Table 8.

(1) $$f_{f}=\frac{\rho_{c}-\rho_{M}}{\rho_{f}-\rho_{M}},$$

where

*f_f_*—fiber volume fraction,

$\rho_{c}$—composite density,

$\rho_{M}$—matrix density,

$\rho_{f}$—fiber density.

The highest fiber volume fraction was observed in the PA6/05 and PBT/05 composites. This result is consistent with expectations, as a lower polymer content with the same amount of fabric leads to a higher fiber volume fraction, which was confirmed by the density measurements. Despite the identical fiber configuration and polymer layer thickness, the PBT-based composites exhibited a lower fiber volume fraction than those made with PA6. This difference may be related to the different processing behavior of the two polymers, including possible differences in wettability and viscosity. However, wettability was not quantified in this study. Therefore, this explanation should be treated as a possible interpretation rather than a confirmed mechanism.

#### 4.1.2. Thermoplastic Fiber Metal Laminate

Analogously to the composites, the density of the TFMLs was determined (Table 9), which enabled further analysis of their mechanical properties in the context of material efficiency. Since the compared laminates contained the same constituents in the same proportions, fiber orientation was not expected to affect density; therefore, in the laminate density analysis, the laminate designation was based on the applied aluminum alloy, polymer type, presence of carbon fiber reinforcement, and polymer content. Here, CF denotes the presence of carbon fiber reinforcement regardless of fiber orientation.

In all laminate configurations, the thinner specimens exhibited higher density than their thicker counterparts. This effect is consistent with that observed in the composites, where a lower polymer content with the same amount of fibers results in a higher proportion of the fibrous phase in the material and, consequently, an increase in density.

### 4.2. Static Mechanical Properties

#### 4.2.1. Base Materials and Thermoplastic Composites

**Figure 5:**
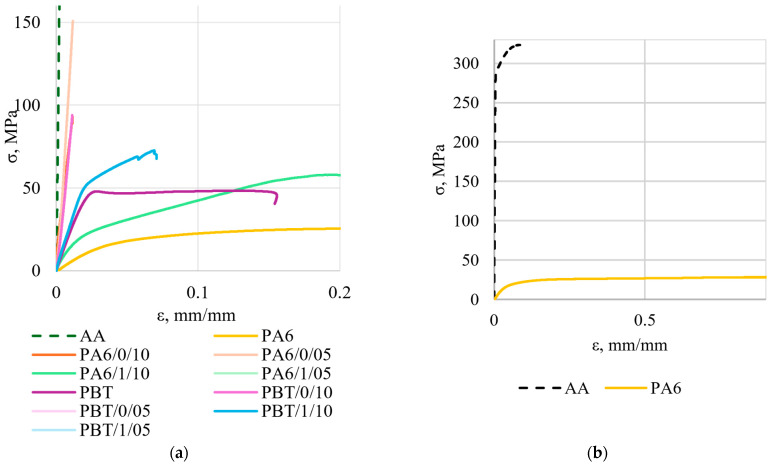
Stress–strain curves of the base materials and CFRTP composites: (**a**) limited strain range; (**b**) full strain range for AA6061 and PA6.

The aluminum alloy AA6061, polymers PA6 and PBT, as well as their corresponding composites, were analyzed. The results for the 1 mm thick PA6- and PBT-based composites, corresponding to the lower fiber content group, were taken from the previous study [52] and are included here as constituent data for the TFML analysis. The results for the 0.5 mm thick composites were obtained in the present study. The obtained results are shown as stress–strain curves in Figure 5a. For clarity, the plots are limited to the range of maximum strength and maximum strain of the composites, while the complete curves illustrating the further behavior of the aluminum alloy and the unreinforced PA6 polymer are presented in Figure 5b.

The analysis of the stress–strain curves of the composites reinforced with fabrics oriented at 0/90° and ±45° reveals significant differences in the deformation behavior and failure mechanisms. For the composites reinforced with 0/90-oriented fabrics (materials PA6/0/10, PA6/0/05, PBT/0/10, and PBT/0/05), a distinct linear region corresponding to the elastic response of the material is observed, followed by an abrupt drop in the curve. A clear knee effect can be identified, characterized by a decrease in stiffness after the initial linear segment, ultimately leading to brittle fracture of the material [71,72,73]. In contrast, the composites reinforced with ±45-oriented fabrics exhibit a completely different mechanical response, with progressive damage evolution resulting in a gradual degradation of stiffness until final failure. This behavior arises from the fact that fibers oriented at ±45° do not carry the load axially in tension but instead rotate and slip relative to the matrix during loading. The complete failure of such composites occurs at higher strain levels compared to the 0/90-oriented ones, and the overall failure process is more gradual in nature.

**Figure 6:**
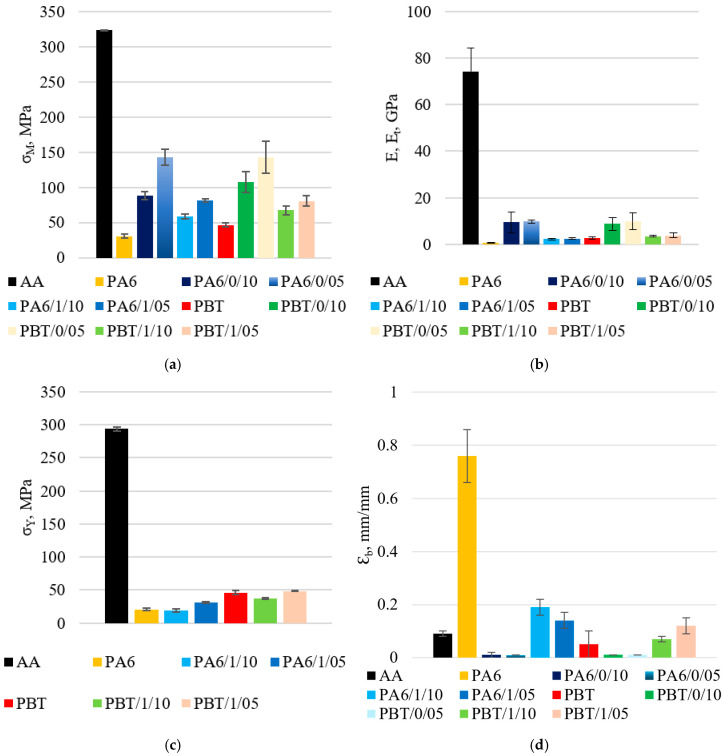
Comparison of the mean mechanical properties of the base materials and CFRTP composites: (**a**) longitudinal elastic modulus E, (**b**) yield strength σ_Y_, (**c**) tensile strength σ_M_, and (**d**) maximum strain at failure Ɛ_b._

Table 10 presents the mean values and standard deviations of the key mechanical properties obtained from the tensile tests, including the longitudinal elastic modulus E, yield strength σ_Y_, tensile strength σ_M_ and elongation at break Ɛ_b_ for the base materials and CFRTP composites. The graphical comparison of these properties is shown in Figure 6, which illustrates the mean values with standard deviations for all analyzed materials.

As shown in Table 10 and Figure 6a, AA6061 exhibited the highest elastic modulus and therefore acted as the main stiffening phase in the TFML structure. The incorporation of carbon fabric markedly increased the stiffness of both polymers, especially in the 0/90° configuration. This effect was stronger in composites with lower polymer content, which is directly related to their higher fiber volume fraction. In contrast, the ±45° configuration showed lower longitudinal stiffness because the fibers were not aligned with the tensile loading direction.

As shown in Table 10 and Figure 6b, AA6061 exhibited the highest yield strength and showed a gradual transition from the elastic to the plastic region. In the composites reinforced with 0/90° fabric, no clear yield point was observed because failure occurred abruptly after the initial linear response. In contrast, the ±45° composites showed a more gradual transition, allowing σ_Y_ to be determined. In this group, PBT-based composites showed higher σ_Y_ values than PA6-based composites.

Tensile strength followed a similar trend to stiffness. The highest composite strengths were obtained for 0/90° fabrics and lower polymer content, while ±45° fabrics gave lower values due to the limited axial contribution of the fibers. Regardless of the matrix type, the addition of carbon fabric clearly improved tensile strength compared with the neat polymers.

The addition of carbon fabric reduced the strain at failure, especially for the 0/90° configuration, where the response was more brittle. The ±45° configuration maintained higher deformability because the inclined fibers allowed shear deformation and progressive fiber rotation during tensile loading.

**Figure 7:**
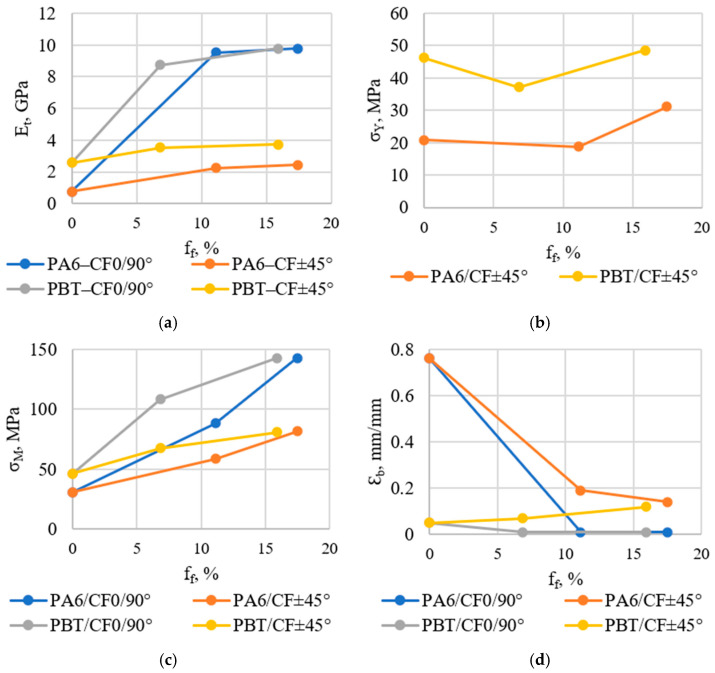
Dependence of the mechanical properties on the carbon fiber volume fraction f_f_ for composites with PA6 and PBT matrix: (**a**) longitudinal elastic modulus E, (**b**) yield strength σ_Y_, (**c**) tensile strength σ_M_, and (**d**) maximum strain at failure Ɛ_b._

The unreinforced PA6 and PBT materials were used as reference points to quantify the effect of carbon fabric reinforcement. Figure 7 presents the relationship between the mechanical properties and the fiber volume fraction *f_f_* for the PA6- and PBT-based composites. To quantify the reinforcing effect, the relative improvement in elastic modulus and tensile strength was calculated with respect to the corresponding neat polymer matrix according to Equation (2):

(2) $$∆P_{c}=\frac{P_{c}-P_{m}}{P_{m}}\cdot 100\%,$$

where:

∆*P_c_*—relative improvement in the selected composite property,

*P_c_*—property of the composite,

*P_m_*—property of the neat polymer matrix

In the PA6-based composites, the 0/90° fabric increased the elastic modulus by approximately 1135–1169% and the tensile strength by approximately 188–365% compared with neat PA6. For the PBT-based composites with 0/90° fabric, the corresponding increases were approximately 240–281% for the modulus and 134–209% for tensile strength. The ±45° configuration was less efficient in improving longitudinal properties. In PA6-based composites, it increased the modulus by approximately 192–216% and the tensile strength by 91–165%, while in PBT-based composites the increases were approximately 37–45% and 46–75%, respectively. These results confirm that the 0/90° configuration is the most efficient for improving tensile stiffness and strength. The higher relative improvement observed for PA6-based composites results mainly from the lower initial properties of the neat PA6 matrix.

The quantitative comparison shows that increasing the fiber volume fraction improves stiffness, but the effect strongly depends on fabric orientation. The 0/90° configuration provides the highest reinforcing efficiency because part of the fiber bundles is aligned with the tensile direction. In contrast, the ±45° configuration mainly promotes shear deformation and fiber rotation, which limits the increase in longitudinal modulus. The higher modulus of neat PBT also explains why the relative increase in stiffness was lower for PBT-based composites than for PA6-based ones.

The effect of fiber volume fraction on yield strength was less direct than for modulus and tensile strength. A clear σ_Y_ value was determined only for the neat polymers and the ±45° composites because the 0/90° composites failed abruptly without a distinct yield point. In the ±45° systems, PBT-based composites showed higher σ_Y_ values than PA6-based composites, which is consistent with the higher yield strength of neat PBT.

Tensile strength increased with fiber volume fraction in all reinforced systems. The effect was strongest for the 0/90° configuration, where the fibers were partially aligned with the loading direction. The ±45° configuration also improved σ_M_ compared with the neat polymers, but the increase was smaller because the tensile response was governed mainly by shear deformation and fiber rotation.

The addition of carbon fabric strongly reduced the strain at failure, especially in the 0/90° configuration. This behavior is typical of fiber-reinforced composites in which the load is transferred to stiff fibers and failure occurs more abruptly. The ±45° composites retained higher deformability because the inclined fibers allowed shear deformation and progressive fiber rotation. In the PBT-based ±45° composites, ε_b_ was higher than in neat PBT because the neat PBT value was recorded at the yield point, according to the adopted testing criterion, whereas the reinforced ±45° structure allowed a more gradual deformation process before failure.

To support the interpretation of the observed differences in the material behavior, a statistical analysis was performed. Because of the limited number of specimens per configuration, the results of the statistical tests should be interpreted with caution. For the purpose of this statistical analysis, the polymer layer thickness factor was excluded, as its effect on the mechanical properties had already been thoroughly examined through the evaluation of the fiber volume fraction. Furthermore, due to the absence of a defined yield point in the composites reinforced with 0/90-oriented fibers, this parameter was analyzed separately. Three mechanical properties were included in the statistical analysis: longitudinal elastic modulus E, tensile strength σ_M_, and strain at failure ε_b_. To verify the assumptions of the parametric tests, the normality of the distribution for each analyzed variable was checked using the Shapiro–Wilk test. The obtained results did not meet the assumption of normal distribution; therefore, the nonparametric Mann–Whitney test was applied. The following null hypothesis was formulated: the type of polymer and the orientation of the reinforcing fabric in the composite do not cause significant differences in the distributions of longitudinal elastic modulus, tensile strength, or strain at failure of the tested materials. The analyses were carried out with a significance level of α = 0.05. The results of the test, presented in Table 11, allow an assessment of statistical significance of the effects of polymer type and fabric orientation on the selected mechanical properties of the composites. *p*-values lower than the assumed significance threshold are marked in bold and indicate statistically significant differences between the compared groups, which in practice confirms the influence of the polymer type or fiber orientation on a given property.

Within the analyzed dataset, the type of polymer did not show a statistically significant effect on the elastic modulus, tensile strength, or maximum strain of the composites. However, this result should be interpreted with caution because the PA6- and PBT-based composites did not have identical fiber volume fractions. Therefore, the effect of polymer type cannot be fully separated from the effect of fiber content in the present composite dataset. In contrast, the fiber orientation in the woven composites significantly affects all the analyzed mechanical properties. As previously discussed, reinforcement with fabric arranged in the 0/90° configuration promotes higher values of the longitudinal elastic modulus E and tensile strength σ_M_ compared to the ±45° orientation. The opposite trend is observed for the maximum strain at failure ε_b_, which shows higher values in the composites reinforced with ±45-oriented fabric. Since the yield point occurred only in the samples with ±45° fiber orientation, the Mann–Whitney test was conducted solely to assess the effect of the polymer type within this group. With a statistical significance of *p*-value = **0.002**, it was confirmed that the PBT-based composites exhibited higher yield strength values than those with PA6. In practice, this means that the PBT-based composites have a higher yield point, and thus a greater ability to carry loads before the onset of permanent deformation, compared to the PA6-based composites. Nevertheless, fiber orientation remains the key factor determining all the analyzed mechanical properties.

#### 4.2.2. Adhesive Films

**Figure 8:**
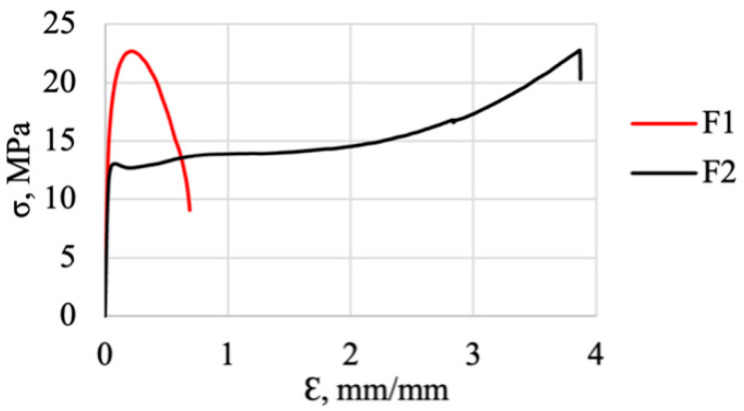
Stress–strain curves of the adhesive films.

To exclude the possibility that the laminates inherit the stiffness and strength characteristics of the adhesive layers, a basic tensile test was also conducted on these materials. The test results are summarized in Table 12, and the stress–strain relationships of these materials are presented in Figure 8.

As shown above, the 23.110 film exhibits a higher elastic modulus compared to FEP, indicating its greater stiffness. Both films reach similar maximum tensile strength values. The purpose of presenting these results was not to conduct a detailed analysis of the films, but rather to demonstrate that the applied adhesive films do not possess high strength properties and therefore do not act as a significant reinforcement for the laminate. Consequently, the overall mechanical properties of the laminates are not determined by the properties of the adhesive films themselves.

#### 4.2.3. Thermoplastic Fiber Metal Laminate

**Figure 9:**
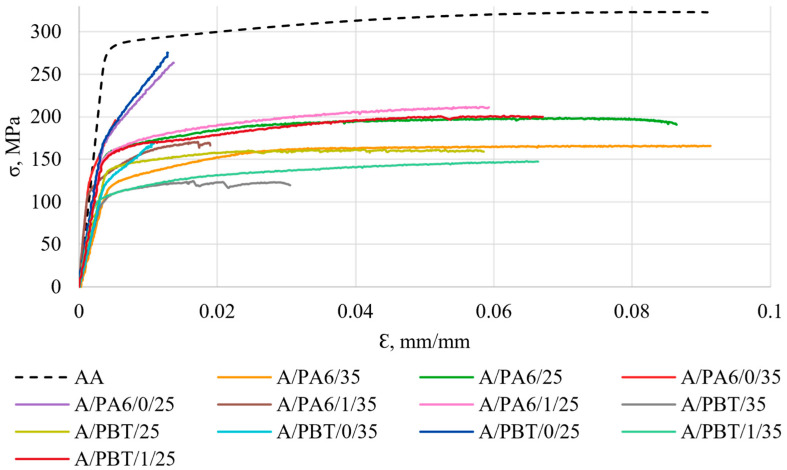
Stress–strain relationships for all tested laminates and aluminum.

After testing the base materials and adhesive films, static tensile tests of the actual laminates were carried out. The stress–strain relationships for all tested laminates and the aluminum alloy are presented in Figure 9.

Table 13 presents the mean values and standard deviations of the basic mechanical properties determined from the static tensile tests: longitudinal elastic modulus E, tensile strength σ_M_, yield strength σ_Y_, and strain at failure Ɛ_b_.

To quantify the effect of laminate configuration, the relative changes in elastic modulus and tensile strength were calculated according to Equation (3):

(3) $$∆P_{c}=\frac{P_{L}-P_{ref}}{P_{ref}}\cdot 100\%,$$

where:

∆*P_l_*—relative change in the selected laminate property,

*P_L_*—property of the analyzed laminate,

*P_ref_*—property of the laminate used as a reference in a given comparison.

For the analysis of the reinforcement effect, the reference laminate was the unreinforced laminate with the same polymer and thickness. For the analysis of the thickness effect, the reference laminate was the corresponding laminate with a total thickness of 3.5 mm.

Reducing the total laminate thickness from 3.5 mm to 2.5 mm generally improved the mechanical properties. In unreinforced PA6-based laminates, the elastic modulus increased from 32.45 GPa to 47.02 GPa, corresponding to an increase of approximately 45%, while tensile strength increased from 164.67 MPa to 191.00 MPa, corresponding to approximately 16%. For unreinforced PBT-based laminates, the modulus increased only slightly, from 32.71 GPa to 33.10 GPa, whereas tensile strength increased from 136.00 MPa to 148.33 MPa, corresponding to approximately 9%. This shows that the effect of thickness reduction was more pronounced in the PA6-based unreinforced laminates.

The introduction of 0/90° carbon fabric clearly improved tensile strength, especially in thinner laminates. Compared with the unreinforced A/PA6/25 laminate, the A/PA6/0/25 laminate showed a tensile strength increase from 191.00 MPa to 268.00 MPa, corresponding to approximately 40%. For the PBT-based system, the increase was even more pronounced: from 148.33 MPa for A/PBT/25 to 269.00 MPa for A/PBT/0/25, corresponding to approximately 81%. In terms of elastic modulus, the same comparison showed a smaller change for PA6-based laminates, from 47.02 GPa to 48.48 GPa, and a stronger increase for PBT-based laminates, from 33.10 GPa to 46.51 GPa.

The ±45° configuration also improved tensile strength, but less effectively than the 0/90° reinforcement. In PA6-based laminates with 2.5 mm thickness, σ_M_ increased from 191.00 MPa for A/PA6/25 to 214.00 MPa for A/PA6/1/25, corresponding to approximately 12%. In PBT-based laminates, σ_M_ increased from 148.33 MPa for A/PBT/25 to 199.67 MPa for A/PBT/1/25, corresponding to approximately 35%. This confirms that the ±45° reinforcement contributes to strength improvement, but its effect is limited by shear-dominated deformation and fiber rotation during tensile loading.

The highest tensile strength values were obtained for the 0/90° reinforced laminates with lower polymer content: A/PA6/0/25 and A/PBT/0/25. Their tensile strengths were almost identical, 268.00 MPa and 269.00 MPa, respectively. This indicates that, in the most favorable 0/90° configuration, the matrix type had a limited effect on maximum tensile strength. However, larger differences between PA6- and PBT-based laminates were observed in unreinforced and ±45° configurations, where the response was more sensitive to matrix behavior, bonding configuration, and deformation mechanisms.

The elastic modulus was governed mainly by the aluminum layers, polymer content, and reinforcement configuration. The highest E values were obtained for thinner laminates and 0/90° reinforcement. The effect of reinforcement was particularly visible in the PBT-based laminates, where E increased from 33.10 GPa for A/PBT/25 to 46.51 GPa for A/PBT/0/25. In PA6-based laminates, the corresponding change was smaller because the unreinforced A/PA6/25 laminate already showed a high modulus value.

Yield strength was affected by polymer content and reinforcement orientation. Thinner laminates generally showed higher σ_Y_ values, which is consistent with the higher relative contribution of the aluminum layers. The highest σ_Y_ values were obtained for 0/90° reinforced laminates with lower polymer content. PA6-based laminates generally showed higher σ_Y_ values than the corresponding PBT-based laminates, but this difference should be interpreted together with the different bonding configuration used for the PBT-based systems.

Tensile strength was controlled mainly by reinforcement and polymer content. The 0/90° reinforced laminates with lower polymer content achieved the highest σ_M_ values, with almost identical strengths for PA6- and PBT-based systems. The ±45° laminates showed lower strength than the 0/90° laminates because their response was governed by shear deformation and fiber rotation. The lowest strengths were obtained for unreinforced laminates, particularly those with higher polymer content. The lower σ_M_ values observed in selected PBT-based TFMLs should not be attributed solely to the intrinsic properties of the PBT matrix or to weak interfacial adhesion. They may result from the combined influence of the bonding configuration, local delamination, processing conditions, and possible differences in thermal history, crystallinity, and residual stresses.

The maximum strain at failure depended mainly on the deformation mechanism of the polymer or composite layer. Unreinforced PA6-based laminates retained the highest deformability, while the 0/90° reinforced laminates failed at the lowest strain values due to their more brittle response. The ±45° configuration allowed higher deformation because shear mechanisms and fiber rotation delayed final fracture.

**Figure 10:**
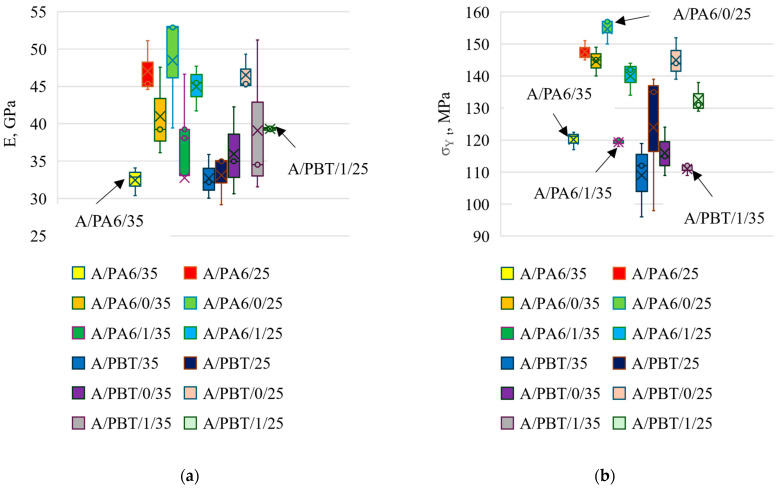

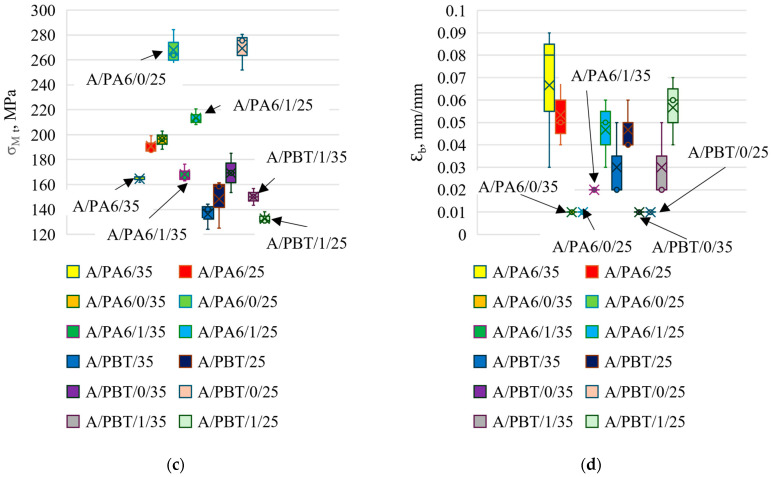
Boxplots of the mechanical properties of TFMLs: (**a**) elastic modulus E, (**b**) yield strength σ_Y_, (**c**) tensile strength σ_M_, (**d**) maximum strain at failure ε_b._

To provide a more comprehensive illustration of the variability in mechanical properties, the analysis was supplemented with a box plot (Figure 10).

The box plots in Figure 10 show that the variability of mechanical properties depended strongly on laminate configuration. The lowest scatter in elastic modulus and strength was generally observed for unreinforced laminates and selected 0/90° reinforced systems, while higher variability was recorded mainly for PBT-based laminates and some ±45° configurations. This indicates that PBT-based systems and off-axis reinforcement were more sensitive to local structural heterogeneity and processing-related defects. The strain at failure showed the highest scatter in unreinforced and ±45° laminates, whereas 0/90° reinforced laminates exhibited very low strain variability due to their more brittle and repeatable fracture behavior. These results show that both mean values and data scatter should be considered when assessing TFMLs for structural applications.

An exploratory statistical analysis of the determined mechanical properties of the laminates was conducted following the procedure described in the Methods section. To analyze the effect of composite configuration on the longitudinal elastic modulus E, the normality of the data distribution was first verified using the Kolmogorov–Smirnov test with Monte Carlo estimation of *p*-values. The assumption of normality was not met for laminates A/PA6/0/25 and A/PBT/25. Levene’s test indicated a lack of homogeneity of variances between groups (with significance *p* = 0.008), which violates one of the key assumptions of classical ANOVA. Therefore, the Kruskal–Wallis test was performed, as it is suitable for cases where the data do not meet the normality assumption and is less sensitive to violations of homogeneity of variance [64]. The following null hypothesis was formulated: changes in polymer type, reinforcement type, and polymer content do not cause significant differences in the E values of the tested materials. The analyses were carried out at a significance level of α = 0.05. *p*-values lower than the established significance threshold were highlighted in bold, indicating the rejection of the null hypothesis and confirming that a given configuration factor (polymer type, reinforcement type, or polymer content) has a statistically significant effect on the E value. Detailed results of the Kruskal–Wallis test for the main effects are presented in Table 14.

The Kruskal–Wallis test analysis showed that the polymer type did not have a statistically significant effect on the longitudinal elastic modulus (*p* = 0.066). However, laminates with PA6 exhibited higher mean ranks than those with PBT, indicating a tendency toward higher E values despite the lack of statistical significance. For the reinforcement factor, a near-significant effect was observed (*p* = 0.044), but detailed post hoc comparisons revealed that only the difference between unreinforced samples and laminates reinforced with 0/90-oriented fabric composites was initially significant (*p* = 0.020). After applying the Bonferroni correction, this difference did not meet the required significance level. The remaining comparisons showed no significant differences (*p* = 0.065 and *p* = 0.419). The strongest effect was found for polymer content, as laminates with a lower polymer fraction exhibited significantly higher E values compared to those with a higher polymer content (*p* = 0.005).

A statistical analysis of the yield strength σ_Y_ was conducted analogously, following the same procedure as for the longitudinal elastic modulus. The assumption of normality was not met for laminates A/PA6/1/35 and A/PBT/1/35 (*p* < 0.001). Levene’s test indicated a lack of homogeneity of variances between groups (*p* = 0.008). Consequently, the Kruskal–Wallis test was applied. The following null hypothesis was formulated: changes in polymer type, reinforcement type, and polymer content do not cause significant differences in the σ_Y_ values of the tested materials. The analyses were carried out at a significance level of α = 0.05. The results of the Kruskal–Wallis test for the main effects are summarized in Table 15. *p*-values lower than the established significance threshold were highlighted in bold, indicating the rejection of the null hypothesis and confirming that a given material configuration factor significantly affects the yield strength σ_Y_.

The Kruskal–Wallis test revealed statistically significant differences in yield strength among laminates with different polymers (*p* = 0.005). Higher σ_Y_ values were recorded for laminates with a PA6 matrix (mean rank 23.42) compared to those with PBT (mean rank 13.58). Polymer content also had a significant effect (*p* < 0.001), with laminates containing thinner polymer layers (mean rank 24.92) exhibiting markedly higher yield strength values than those with thicker matrix layers (mean rank 12.08). The reinforcement type was another influential factor (*p* = 0.040). The highest yield strength values were observed in laminates reinforced with fibers oriented at 0/90° (mean rank 24.75), followed by unreinforced systems (mean rank 16.00), while the lowest values were recorded for laminates with ±45° fiber orientation (mean rank 14.75). Post hoc Mann–Whitney tests confirmed that laminates reinforced with 0/90° fibers achieved significantly higher yield strength than unreinforced laminates (*p* = 0.049), and an even greater difference was observed compared to laminates reinforced with ±45° fibers (*p* = 0.018).

The next stage involved the statistical analysis of tensile strength. In all cases, the data followed a normal distribution (*p* > 0.05). For all analyzed factors, Levene’s test showed no reason to reject the null hypothesis of equal variances (*p* > 0.05). Therefore, for the analysis of σ_M_ in laminates, since the assumptions were met, it was possible to apply the classical one-way analysis of variance (ANOVA). The following null hypothesis was formulated: changes in polymer type, reinforcement type, and polymer content do not cause significant differences in the σ_M_ values of the tested materials. The analyses were performed at a significance level of α = 0.05. The results of the ANOVA are summarized in Table 16. *p*-values lower than the established significance threshold were highlighted in bold, indicating the rejection of the null hypothesis and confirming that polymer type, fiber orientation, or polymer content significantly affect the tensile strength of the laminates.

The analysis showed that the polymer type did not have a statistically significant effect on tensile strength. In contrast, both the reinforcement type and polymer content were found to be statistically significant factors. The analysis of mean values revealed that laminates with lower polymer content (2.5 mm) achieved higher tensile strength values compared to those with thicker polymer layers (3.5 mm). Since the reinforcement type factor included more than two groups (no reinforcement, 0/90°, ±45°), additional post hoc Tukey HSD tests were performed to identify which groups differed significantly. The results indicated that significant differences occurred between unreinforced laminates and those reinforced with 0/90-oriented fabric (*p* = 0.012), as well as between unreinforced laminates and those reinforced with ±45° fabric (*p* < 0.001). However, the difference between the 0/90° and ±45° reinforced laminates was not statistically significant (*p* = 0.246). This suggests that the key factor influencing tensile strength is the presence of reinforcement itself, which substantially increases the tensile strength of laminates compared to unreinforced samples, while the specific fiber orientation (0/90° or ±45°) does not significantly affect the results.

The final stage involved the statistical analysis of maximum strain at failure. In all cases, the data followed a normal distribution. As with the other mechanical properties recorded during the static tensile tests of laminates, Levene’s test revealed significant heterogeneity of variances between groups in the mean-based tests, indicating that the assumption of homogeneity was not met. In accordance with the previously adopted approach, Welch’s ANOVA was applied (Table 17), along with the Tamhane post hoc test. The following null hypothesis was formulated: changes in polymer type, reinforcement type, and polymer content do not cause significant differences in the ε_b_ values of the tested materials. The analyses were carried out at a significance level of α = 0.05. *p*-values lower than the established significance threshold were highlighted in bold, indicating the rejection of the null hypothesis and confirming that polymer type, fiber orientation, or polymer content significantly affect the strain at failure of the laminates.

The Welch ANOVA analysis revealed a significant effect of the reinforcement type on the maximum strain at failure. Further post hoc analysis showed that the maximum strain at failure differed significantly between unreinforced samples and those reinforced with fibers in the 0/90° configuration (*p* < 0.001), as well as between the ±45° and 0/90° configurations (*p* < 0.001). The difference between unreinforced samples and those with the ±45° configuration was not statistically significant (*p* = 0.515). These results indicate that introducing fibers in the 0/90° configuration significantly limits the deformability of the composites, leading to more brittle failure behavior, while the ±45° configuration maintains a deformation capacity similar to that of the polymer matrix.

Based on the measured density values and the determined longitudinal elastic modulus and tensile strength, the specific longitudinal modulus E_spec_ and specific strength σ_spec_ were calculated. The specific modulus is defined as the ratio of the elastic modulus to the material density (Equation 4), while the specific strength is defined as the ratio of tensile strength to density (Equation 5) [74]. These parameters enable the comparison of materials in terms of their stiffness and strength relative to mass, which is particularly important in industrial applications [75], where weight reduction in structures is a key consideration. The calculated indicators are summarized in Table 18.

(4) $$E_{spec}=\frac{E}{\rho }$$

(5) $$\sigma_{spec}=\frac{\sigma_{M}}{\rho }$$

where:

– *E_spec_*—specific modulus,

– σ*_spec_*—specific strength,

– *E*—longitudinal elastic modulus,

– σ*_M_*—tensile strength,

– *ρ*—density.

**Figure 11:**
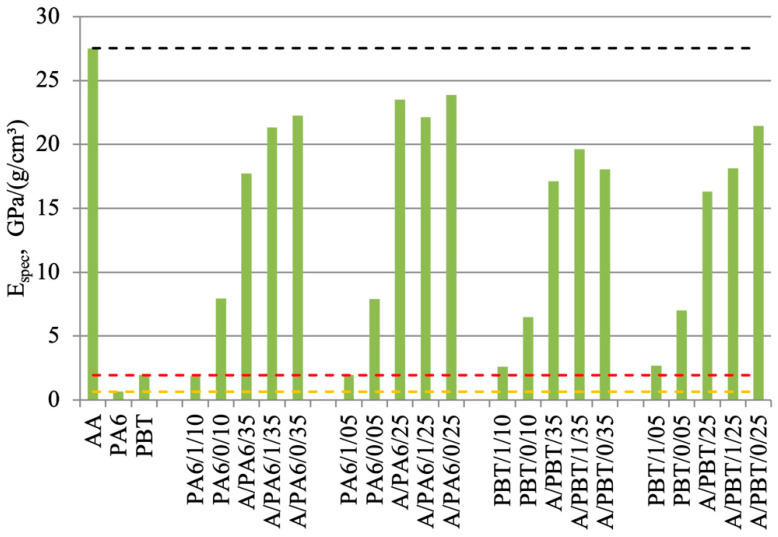
Specific modulus of the tested materials in relation to the reference values of the base materials: AA (black dashed line), PA6 (orange dashed line), and PBT (red dashed line).

**Figure 12:**
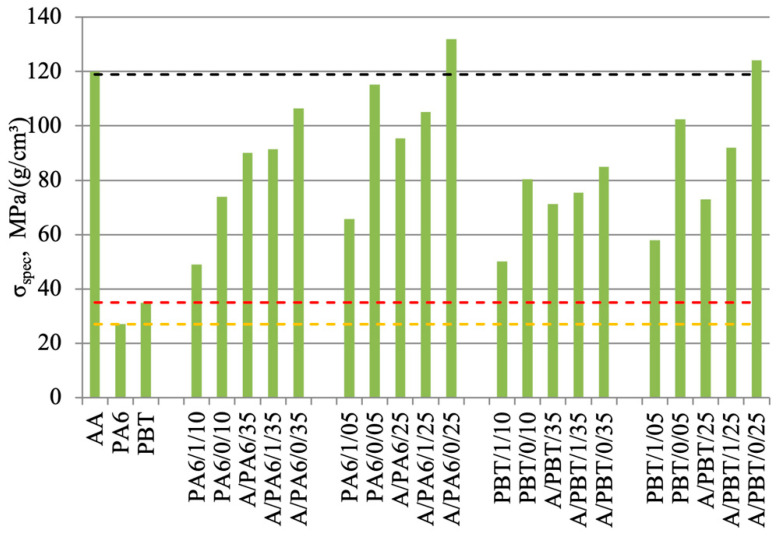
Specific strength of the tested materials in relation to the reference values of the base materials: AA (black dashed line), PA6 (orange dashed line), and PBT (red dashed line).

For a more comprehensive comparative analysis, these results were also presented graphically in Figure 11 and Figure 12, where dashed lines indicate the reference values of the base materials, allowing the behavior of the composites and laminates to be compared with their constituent components.

The specific property analysis confirms that carbon fabric reinforcement substantially improves the stiffness- and strength-to-weight efficiency of both polymers. The strongest effect was observed for the 0/90° composites, especially those with lower polymer content, while the ±45° configuration gave lower specific values due to the less favorable fiber alignment with the tensile direction. This confirms that the reinforcement orientation, rather than the matrix type alone, governs the mass-normalized mechanical efficiency of the composites.

Among the TFMLs, the best mass-normalized performance was obtained for thinner laminates reinforced with 0/90° fabric. The A/PA6/0/25 and A/PBT/0/25 laminates reached specific strength values comparable to or higher than aluminum, confirming the potential of the developed TFML structures for lightweight applications. The lower values obtained for unreinforced and ±45° laminates show that efficient load transfer requires both a favorable fiber orientation and a reduced polymer core thickness.

### 4.3. Modeling the Mechanical Properties of the Laminate Using the Superposition Method

**Figure 13:**
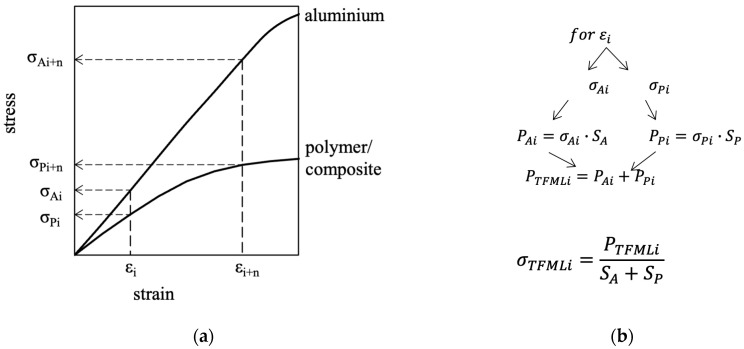
Analysis of stresses and forces in the aluminum and polymer/composite layers for instantaneous strain values: (**a**) stress–strain relationship for individual layers, (**b**) force equilibrium and laminate stress calculation.

The superposition method was employed in this study to analyze the mechanical response of TFMLs based on the properties of the base materials and CFRTP composites. Its application made it possible to compare model-predicted stress–strain curves with experimental results and to evaluate the influence of polymer type, polymer content within the TFML structure, and fiber bundle orientation in the fabric on the agreement between the model and the actual behavior. The model discussion was focused on fiber-reinforced TFMLs, while the unreinforced polymer–aluminum laminates were produced and tested only as reference systems to assess how the introduction of the fiber-reinforced composite layer affects the overall mechanical response. In the applied method, the average stress values for the aluminum layers of the TFMLs σ_Ai_ and for the polymer layers σ_Pi_ were first determined under equal strain values ε_i_ (Figure 13a). Knowing the stress values at equal strains σ_Ai_ and σ_Pi_ and the cross-sectional areas of the respective layers S_A_ and S_P_, the forces carried by these layers PAi and PPi were calculated. The total force PTFMLi carried by the laminate at a given strain εi was then obtained as the sum of the forces transmitted through each layer. The schematic representation of this method is shown in Figure 13b.

**Figure 14:**
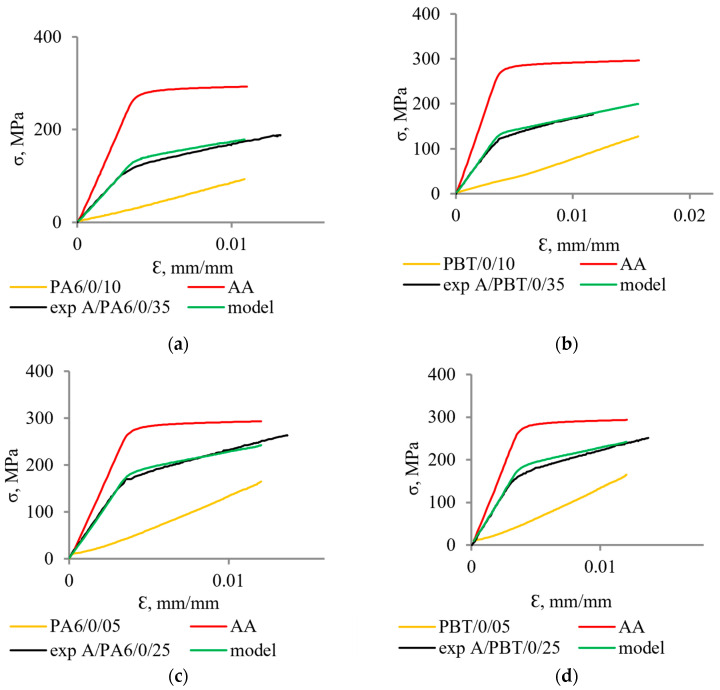

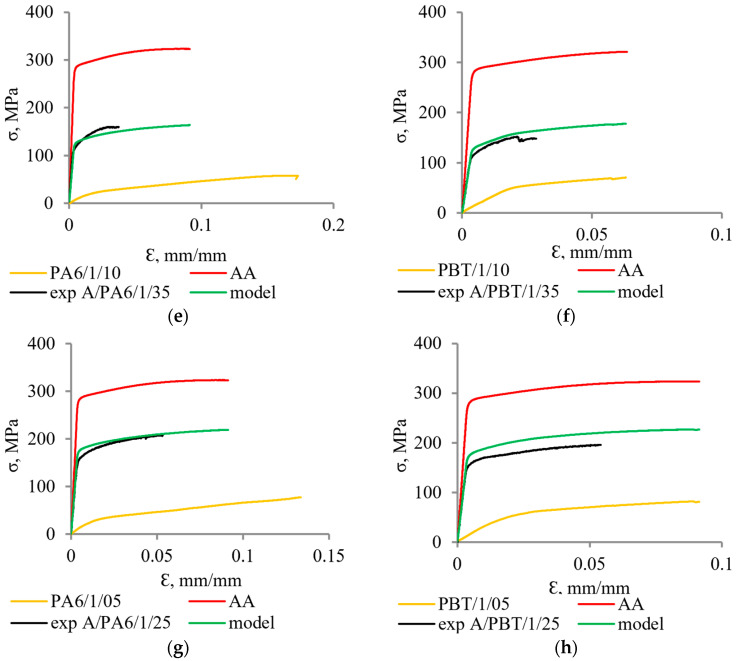
Fitting of the superposition model to the stress–strain curves of the laminates: (**a**,**b**) reinforced with 0/90° fabric and lower fiber content, (**c**,**d**) reinforced with 0/90° fabric and higher fiber content, (**e**,**f**) reinforced with ±45° fabric and lower fiber content, (**g**,**h**) reinforced with ±45° fabric and higher fiber content.

After determining the values of the forces P_TFMLi_ for successive strain levels ε_i_, a theoretical force–strain curve for the layered composite was obtained. By calculating the average stresses σ_TFMLi_ in the cross-section of the tensile specimen at each strain level ε_i_, the stress–strain curves for the individual TFMLs were generated. Figure 14 presents a comparison of the experimentally obtained stress–strain curves for the constituent materials and the laminates, together with the corresponding modeling results.

To quantitatively evaluate the agreement between the experimental and model-predicted mean elastic modulus values, the absolute percentage error was calculated for each laminate configuration according to Equation (6). The calculated *APE* values are presented in Table 19.

(6) $${APE}_{i}=\left|\frac{A_{i}-F_{i}}{A_{i}}\right|\cdot 100\%,$$

where:

– *APE*—absolute percentage error, %,

– *A_i_*—mean experimental elastic modulus for *i*-th laminate configuration, GPa,

– *F_i_*—model-predicted elastic modulus for the *i*-th laminate configuration, GPa,

– *i*—number of the analyzed laminate configuration.

The comparison shown in Figure 14 and Table 19 indicates that the agreement between the experimental and model-predicted elastic modulus values depends on the laminate configuration. The lowest APE values were obtained for the 0/90° reinforced laminates with lower polymer content, namely A/PA6/0/25 and A/PBT/0/25, for which the error was 0.68% and 1.03%, respectively. This confirms that the superposition model provides the best agreement for configurations dominated by axial load transfer and a stable elastic response. For the remaining 0/90° reinforced laminates, the APE values were also relatively low, reaching 7.49% for A/PA6/0/35 and 5.22% for A/PBT/0/35. Higher discrepancies were observed for laminates reinforced with ±45° fabric. In this group, the APE values ranged from 3.44% for A/PA6/1/25 to 24.25% for A/PBT/1/25. The larger error in selected ±45° configurations results from the fact that their response is more strongly affected by shear deformation, fiber rotation, local delamination, and progressive stiffness degradation, which are not included in the simplified superposition model. Therefore, the model should be treated primarily as an approach for estimating the elastic stiffness of TFMLs rather than as a complete description of the full stress–strain response or damage evolution.

Overall, the APE analysis confirms that the superposition method can reasonably predict the elastic modulus of TFMLs, especially for 0/90° reinforced laminates. At the same time, the higher errors observed for selected ±45° and PBT-based configurations indicate the limitations of the model in cases where the laminate response is governed by nonlinear deformation mechanisms and interfacial effects.

For PBT-based laminates, deviations from the model appeared earlier than in PA6-based ones, which may be associated with local delamination, the applied bonding configuration, and deformation mechanisms not captured by the superposition model.

### 4.4. Fractography

This section presents an analysis of the damage mechanisms and fracture morphology following static tensile tests. The evaluation was carried out separately for composites and laminates, taking into account the influence of the polymer matrix type and content, as well as the type of reinforcement.

#### 4.4.1. Thermoplastic Composites

**Figure 15:**
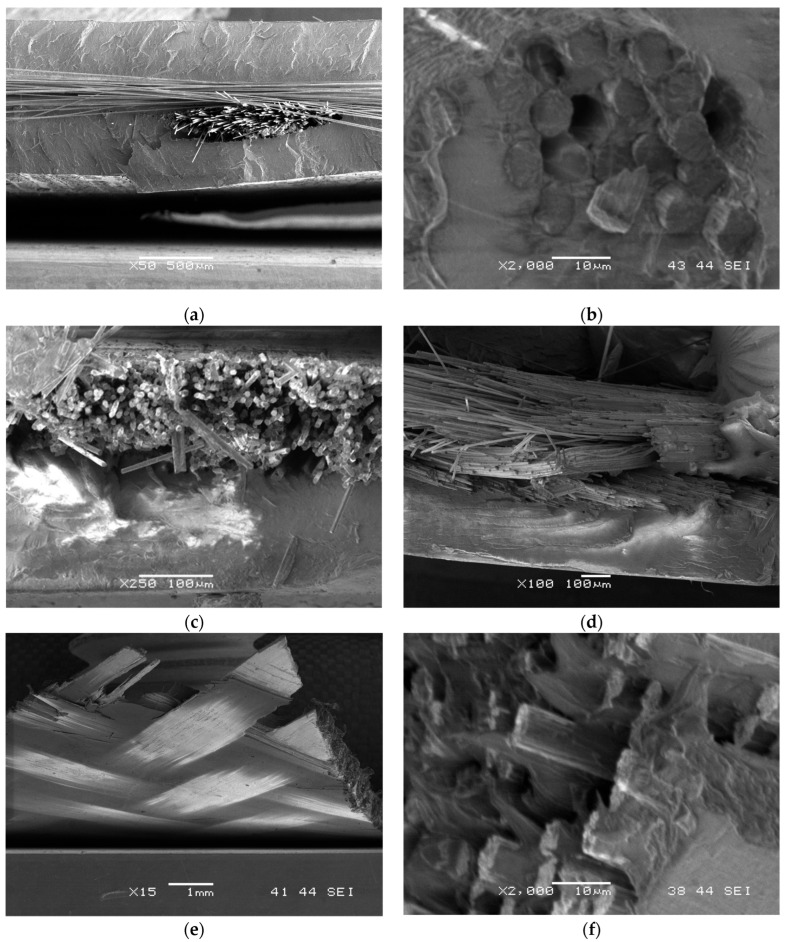

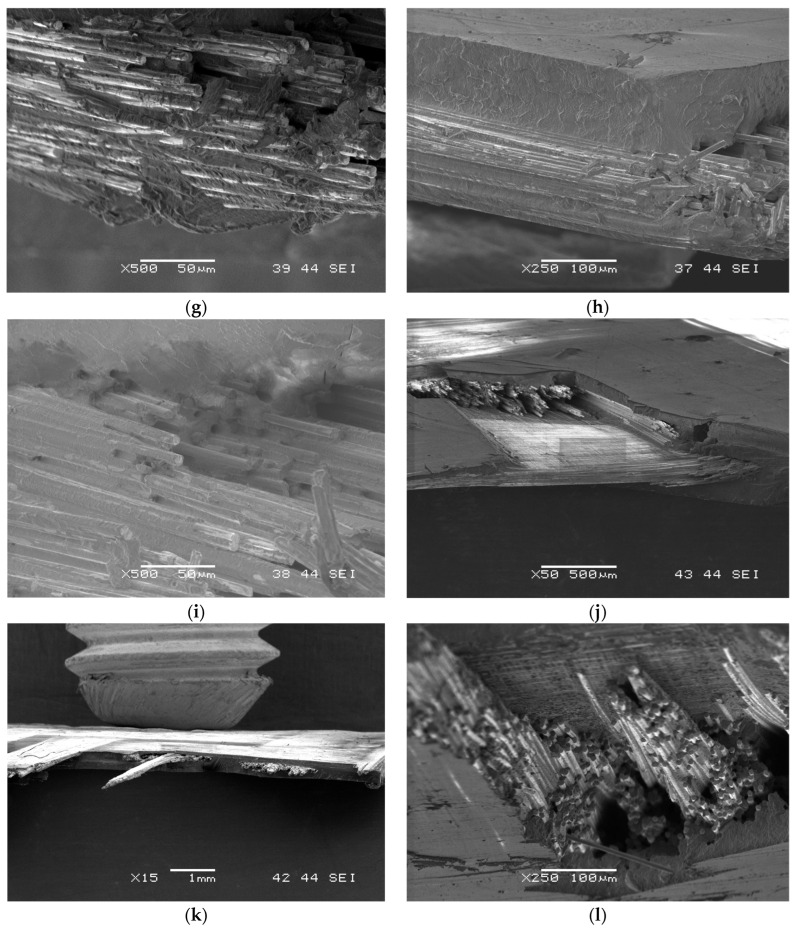
Failure features observed on the fracture surfaces: (**a**) composite reinforced with fabric oriented at 0/90°, exhibiting brittle fracture characteristics, (**b**) fibers pulled out from the matrix and devoid of polymer, with visible voids near fiber bundles, (**c**) local separation between fabric-rich and matrix-rich regions, (**d**) areas between fibers not fully filled by the polymer matrix, (**e**) varied surface topography with exposed fiber bundles oriented at ±45°, (**f**) broken fibers, (**g**) local areas with exposed fibers and limited matrix coverage, (**h**) local separation along the fiber–matrix region, (**i**) fiber pull-out, (**j**) locally detached fiber bundle from the matrix, (**k**) local fracture irregularity, (**l**) fiber breakage.

A detailed description of the damage observed for each of the analyzed composite base materials is provided in Table 20, while Figure 15 presents selected characteristic forms of failure.

The fracture analysis of the composites revealed that the most common failure mechanism was fiber pull-out from the matrix. Similar mechanisms have been reported in other studies on thermoplastic composites reinforced with carbon fabric [47,76,77]. The fibers were often broken or sharply fractured, and in many cases, their surfaces were free of matrix residue, which is consistent with observations described in [45]. Local voids between fibers were also observed, which may indicate incomplete impregnation of the composite and locally limited matrix–fiber contact [78]. Composites reinforced with fabric oriented at ±45° exhibited markedly more irregular fracture surfaces, whereas those with 0/90° orientation showed a more ordered fracture morphology, dominated by perpendicular fiber bundles and less developed damage zones. This trend was observed for both PA6- and PBT-based composites, regardless of polymer content. Reinforcement with ±45° fabric led to complex failure mechanisms involving shear, while in 0/90° configurations, cracking occurred mainly along the matrix with a simpler separation mechanism. Similar relationships between fiber orientation and fracture behavior have been reported in other works [79], confirming the general consistency of this phenomenon. Composites with a lower polymer content exhibited more irregular fractures and pronounced delamination, whereas those with a higher polymer content displayed denser fracture patterns and more distinct phase separation. Thinner matrices tended to promote local stress concentrations and point-like damage, while thicker matrices acted as stabilizing layers but facilitated crack propagation over a larger area. Both thermoplastics exhibited similar damage characteristics within the same reinforcement configurations.

#### 4.4.2. Thermoplastic Fiber Metal Laminate

Subsequently, after conducting microscopic observations of the fracture surfaces, the main morphological features and failure mechanisms in the TFMLs after the tensile test were identified. Table 21 summarizes the most frequently observed phenomena visible in the SEM images of selected laminates.

**Figure 16:**
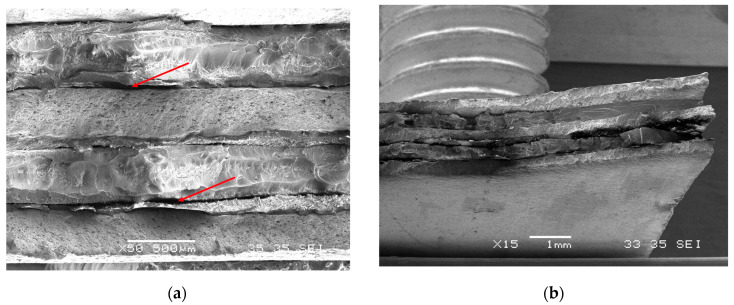

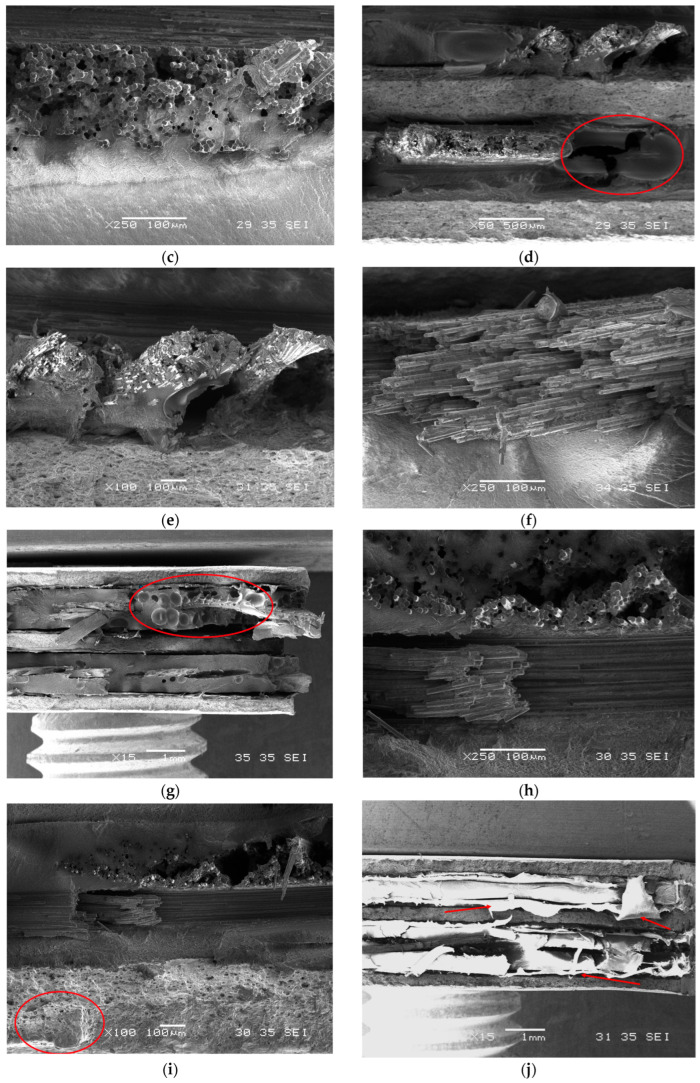

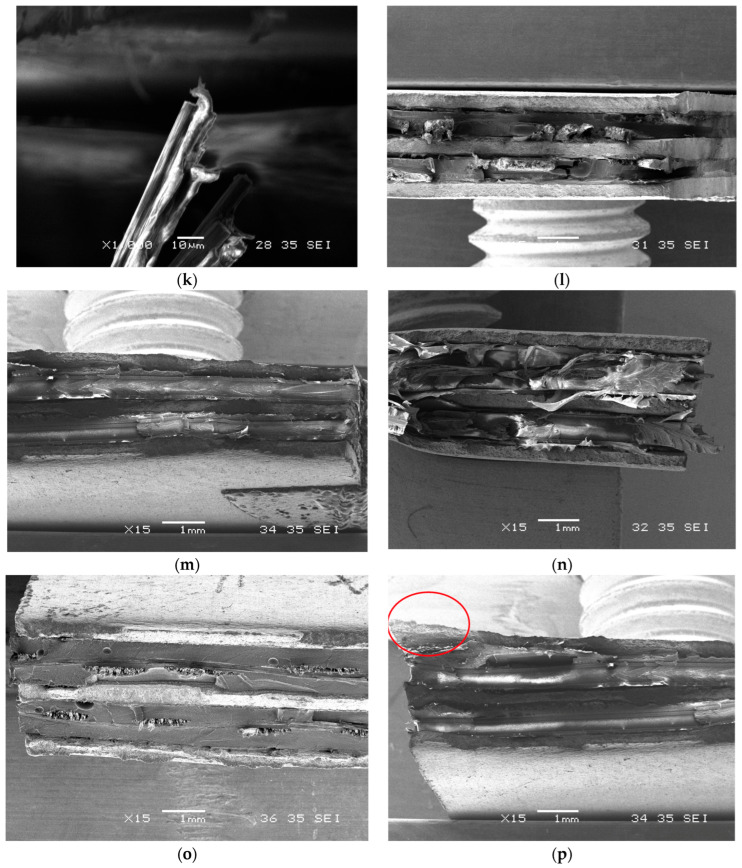
Failure modes observed on the fracture surfaces of TFMLs: (**a**) layer separation (marked in red) and visible dimple fracture in the central metal layer, (**b**) nonlinear metal cracking path and exposed aluminum area without polymer coverage, (**c**) pulled-out fibers and traces of single fiber pull-outs, (**d**) matrix Fcracking at the site of an air bubble (indicated with a red ellipse), (**e**) fiber bundles torn out of the matrix, (**f**) broken fibers of varying lengths, (**g**) numerous air voids, (**h**) visible fiber arrangement in the 0/90° system—fibers at 0° partially broken with distinct traces of bundle pull-out, while 90° fiber bundles are completely pulled out, leaving voids in the matrix, (**i**) a cavity resembling a dimple fracture (marked in red), (**j**) torn and heavily deformed adhesive film (indicated with red arrows), (**k**) exposed fiber devoid of polymer matrix, (**l**) smooth fracture surface of the central aluminum layer, (**m**) irregular crack morphology, (**n**) sharp and irregular polymer fracture edges, (**o**) smooth yet irregular fracture surfaces of aluminum layers, (**p**) aluminum edge of the “torn edge” type (marked in red).

Representative SEM images illustrating the phenomena described in the table are presented in Figure 16.

Regardless of the polymer type, fabric orientation, or reinforcement content, the dominant failure mechanism in all TFMLs was layer separation and delamination at the metal–composite interface. Crack propagation was frequently observed along the metal–composite boundary rather than through the constituent layers, suggesting that the interfacial region played an important role in the failure process. This process was often accompanied by fiber pull-out and localized matrix cracking. In most samples, traces of plastic deformation of the aluminum layers and local dimple fracture depressions were also observed, indicating the contribution of plastic deformation mechanisms to the overall failure process. This phenomenon has also been confirmed in other studies [80], which demonstrated that plastic deformation of the metallic layers plays a key role in energy absorption in FML structures. In laminates reinforced with 0/90° woven fabric, the fracture surfaces were more ordered and flatter, with cracking predominantly occurring along the fiber direction (similar to the behavior observed in 0/90° CFRTP composites). The failure mechanism exhibited an axial character, with a limited number of local debonding zones and low surface roughness of the fracture. Fiber breakage along the loading direction and distinct imprints of fiber bundles in the matrix were frequently observed. The aluminum layers showed predominantly smooth fracture surfaces with a relatively uniform morphology. In laminates reinforced with ±45° woven fabric, the fracture surfaces were noticeably more irregular, confirming previous observations that the fracture morphology of TFMLs reflects the failure characteristics of the composite constituent layer. The fracture exhibited an asymmetric character, with local variations in height and crack propagation direction. In the aluminum layers, torn edge features were observed, indicating the contribution of shear mechanisms to the overall failure process. In laminates with lower fiber content and a higher proportion of the polymer core, air voids (particularly in laminates with PA6), local matrix losses, and traces of plastic deformation of the matrix were more frequently observed. Air voids are classified as defects arising during the manufacturing process [45]. The fracture surfaces exhibited a more irregular morphology, and the delamination zones were more extensive, especially in laminates with PBT. In contrast, laminates with higher fiber content and thinner polymer cores were characterized by fiber breakage, flatter and more ordered fracture surfaces, and a lower number of defects in the form of air voids.

## 5. Conclusions

The study developed and characterized thermoplastic fiber metal laminates (TFMLs) based on aluminum alloy and thermoplastic composites reinforced with carbon fabric using PA6 and PBT matrices. The applied hot-pressing method enabled the fabrication of PA6- and PBT-based TFMLs; however, the metal–composite interface remained the critical region controlling the failure process. The newly developed laminates with a PBT matrix were compared to the previously developed and characterized PA6-based laminates to evaluate the influence of polymer type, fiber orientation, and fiber content on the mechanical properties and failure mechanisms.

– The results showed that laminates with a higher fiber content exhibited greater stiffness, yield strength, and tensile strength. In both PA6- and PBT-based laminates, reinforcement with fabric oriented at 0/90° provided the most efficient load transfer. At a fiber volume fraction of approximately 16–18% in the composite layer, the laminates achieved specific strength values comparable to those of aluminum, confirming the high efficiency of the developed TFML structure.
– Modeling based on the superposition method accurately reproduced the laminate response in the elastic region, especially for systems reinforced with 0/90° fabric, confirming the applicability of this approach for predicting laminate properties from their constituent materials.
– Laminates with a PA6 matrix exhibited lower variability in elastic modulus, yield strength, and tensile strength, indicating higher mechanical stability. PBT-based laminates achieved comparable specific strength values in selected configurations and showed more frequent interfacial separation and delamination. However, the adhesion quality could not be quantitatively compared based on the present SEM observations alone. Moreover, because a different bonding configuration was required for the PBT-based laminates, the effect of the adhesive system could not be separated from the effect of the polymer matrix. Considering that TFMLs with PBT achieved mechanical parameters close to those with PA6, the selection of the polymer should depend on service conditions and the operating environment. Nevertheless, given the excellent intrinsic properties of PBT-based composites, further improvement of the bonding methods between this polymer and metallic layers is recommended.
– Microscopic analysis indicated that the dominant visible damage mechanism in the laminates was delamination at the metal–composite interface. The intensity and morphology of this phenomenon depended on the polymer type and the processing conditions during hot pressing. The fracture morphology of the TFMLs reflected the fiber bundle architecture in the composite layer, demonstrating a close relationship between the micromechanical failure mechanism of the composite and the overall structural behavior of the TFML.
– The conducted research confirmed the potential of thermoplastic fiber metal laminates as structural materials with a favorable strength-to-weight ratio. The obtained results indicate that further development of these structures should focus on improving and quantitatively evaluating the metal–composite interface, as well as increasing the fiber content in the composite base materials. The environmental resistance and dimensional stability of PBT were considered only as literature-based motivation for material selection and should be verified in future dedicated tests. Future work should include peel and lap shear tests, as well as fatigue and environmental testing, to quantitatively evaluate the metal–composite interface and more comprehensively assess the durability and potential application areas of TFMLs.

## 6. Patents

Pat.248395.

## Figures and Tables

**Table 1:** Nominal properties of the polymer base materials according to supplier data.

Property	PA6	PBT
Density, g/cm^3^	1.12	1.31
Young’s modulus, MPa	2500–3500	2900
Tensile strength, MPa	65–85	60
Elongation at break, %	10–100	200

**Table 2:** Properties of carbon fibers.

Type of Carbon Fiber	T300
Number of filaments	3000
Density, g/cm^3^	1.76
Filament diameter, mm	0.007
Tensile strength, MPa	3530
Elastic modulus, GPa	230
Elongation at break, %	1.5

**Table 3:** Properties of carbon fabric.

Type	Areal Density	Thickness, mm	Warp Density	Weft Density
TWILL 2/2	160 g/m^2^	0.34	400	400/m

**Table 4:** Properties of the aluminum alloy.

Property	AA6061
Density, g/cm^3^	2.7
Young’s modulus, GPa	68.9
Yield strength, MPa	270–287
Tensile strength, MPa	307–323
Elongation at break, %	10.4–11.2

**Table 5:** Technological pressing parameters for TFMLs.

Material	Temperature, °C	Pressure, MPa	Process Time, s
PA6-based laminates	220	no pressure	120
		2	60
		5	60
		20	480
PBT-based laminates	225	no pressure	120
		2	60
		5	60
		20	480

**Table 6:** List of investigated materials and their designations.

Material	Designation
AA6061	AA
Adhesive film 23.110	F1
Adhesive film FEP	F2
PA6	PA6
PA6–CF0/90°, b = 1 mm	PA6/0/10
PA6–CF0/90°, b = 0.5 mm	PA6/0/05
PA6–CF ± 45°, b = 1 mm	PA6/1/10
PA6–CF ± 45°, b = 0.5 mm	PA6/1/05
PBT	PBT
PBT–CF0/90°, b = 1 mm	PBT/0/10
PBT–CF0/90°, b = 0.5 mm	PBT/0/05
PBT–CF ± 45°, b = 1 mm	PBT/1/10
PBT–CF ± 45°, b = 0.5 mm	PBT/1/05
AA6061–PA6, b = 3.5 mm	A/PA6/35
AA6061–PA6, b = 2.5 mm	A/PA6/25
AA6061–PA6–CF0/90°, b = 3.5 mm	A/PA6/0/35
AA6061–PA6–CF0/90°, b = 2.5 mm	A/PA6/0/25
AA6061–PA6–CF ± 45°, b = 3.5 mm	A/PA6/1/35
AA6061–PA6–CF ± 45°, b = 2.5 mm	A/PA6/1/25
AA6061–PBT, b = 3.5 mm	A/PBT/35
AA6061–PBT, b = 2.5 mm	A/PBT/25
AA6061–PBT–CF0/90°, b = 3.5 mm	A/PBT/0/35
AA6061–PBT–CF0/90°, b = 2.5 mm	A/PBT/0/25
AA6061–PBT–CF ± 45°, b = 3.5 mm	A/PBT/1/35
AA6061–PBT–CF ± 45°, b = 2.5 mm	A/PBT/1/25

**Table 7:** Density measurements of polymer base materials and CFRTP composites.

	ρ, g/cm^3^	
Material	$\bar{x}$	s
PA6	1.13	0.01
PA6/10	1.20	0.01
PA6/05	1.24	0.00
PBT	1.32	0.01
PBT/10	1.35	0.01
PBT/05	1.39	0.01

**Table 8:** Fiber volume fraction in the composites.

Composite	*f_f_*, %
PA6/10	11.11
PA6/05	17.46
PBT/10	6.82
PBT/05	15.91

**Table 9:** Density measurements of thermoplastic fiber metal laminates.

	ρ, g/cm^3^	
Laminate	$\bar{x}$	s
A/PA6/35	1.83	0.02
A/PA6/25	2.00	0.03
A/PA6/CF/35	1.84	0.02
A/PA6/CF/25	2.03	0.05
A/PBT/35	1.91	0.03
A/PBT/25	2.03	0.09
A/PBT/CF/35	1.99	0.02
A/PBT/CF/25	2.17	0.02

**Table 10:** Mechanical properties obtained from static tensile tests of base materials and CFRTP composites.

	E, GPa		σ_Y_, MPa		σ_M_, MPa		Ɛ_b_, mm/mm	
	$\bar{x}$	s	$\bar{x}$	s	$\bar{x}$	s	$\bar{x}$	s
AA	74.32	9.93	293.56	2.98	323.64	0.31	0.09	0.01
PA6	0.77	0.14	20.80	1.92	30.67	2.68	0.76	0.10
PA6/0/10	9.51	4.41	–		88.24	5.35	0.01	0.01
PA6/0/05	9.77	0.59			142.78	11.40	0.01	0.00
PA6/1/10	2.25	0.36	18.80	2.28	58.58	3.71	0.19	0.03
PA6/1/05	2.43	0.39	31.33	0.58	81.32	1.76	0.14	0.03
PBT	2.57	0.50	46.00	3.16	46.22	2.97	0.05	0.05
PBT/0/10	8.75	2.66	–		108.04	14.73	0.01	0.00
PBT/0/05	9.78	3.59			142.62	22.43	0.01	0.00
PBT/1/10	3.53	0.21	37.00	1.00	67.42	6.00	0.07	0.01
PBT/1/05	3.73	1.03	48.67	1.15	80.70	7.74	0.12	0.03

Note: “–” indicates that the yield strength was not determined due to the absence of a distinct yield point.

**Table 11:** *p*-values for the effect of polymer type and fabric orientation on the mechanical properties of the composites.

	*p*-Value		
	E	σ_M_	Ɛ_b_
Polymer	0.208	0.183	0.140
Fabric orientation	**<0.001**	**<0.001**	**<0.001**

**Table 12:** Mechanical properties of the adhesive films.

Property		Film	
		F1	F2
E, MPa	1	444.21	211.80
	2	553.01	282.61
	3	452.10	223.19
	$\bar{x}$	483.11	239.20
	s	60.67	38.02
σ_M_, MPa	1	22.74	12.43
	2	20.01	22.80
	3	22.66	21.43
	$\bar{x}$	21.80	18.89
	s	1.55	5.63

**Table 13:** Mechanical properties of TFMLs.

	E, GPa		σ_Y_, MPa		σ_M_, MPa		Ɛ_b_, mm/mm	
	$\bar{x}$	s	$\bar{x}$	s	$\bar{x}$	s	$\bar{x}$	s
A/PA6/35	32.45	1.88	120.33	3.06	164.67	2.31	0.07	0.03
A/PA6/25	47.02	3.57	148.00	3.00	191.00	7.00	0.06	0.01
A/PA6/0/35	40.98	5.91	144.67	4.51	195.67	7.51	0.01	0.00
A/PA6/0/25	48.48	7.85	154.67	4.04	268.00	14.42	0.01	0.00
A/PA6/1/35	39.26	6.87	119.33	1.15	168.33	6.81	0.02	0.00
A/PA6/1/25	44.96	3.02	140.00	5.29	214.00	6.56	0.05	0.01
A/PBT/35	32.71	2.94	109.00	11.79	136.00	10.58	0.03	0.02
A/PBT/25	33.10	3.39	124.00	22.61	148.33	20.23	0.05	0.01
A/PBT/0/35	35.96	5.88	116.00	7.55	169.33	15.50	0.01	0.00
A/PBT/0/25	46.51	2.43	145.00	6.56	269.00	14.93	0.01	0.00
A/PBT/1/35	39.10	10.61	111.00	1.73	150.33	6.51	0.03	0.01
A/PBT/1/25	39.31	0.36	132.67	4.73	199.67	2.31	0.06	0.01

**Table 14:** *p*-values for main effects obtained from the Kruskal–Wallis test for modulus E.

Effect	Rank	*p*-Value
Polymer type	PA6—21.72 PBT—15.28	0.066
Reinforcement	no reinforcement—12.54 0/90°—22.96 ±45°—20.00	**0.044**
Polymer content	3.5 mm—13.58 2.5 mm—23.42	**0.005**

**Table 15:** *p*-values for main effects obtained from the Kruskal–Wallis test for σ_Y_.

Effect	Rank	*p*-Value
Polymer type	PA6—23.42 PBT—13.58	**0.005**
Reinforcement	no reinforcement—16.00 0/90°—24.75 ±45°—14.75	**0.040**
Polymer content	3.5 mm—12.08 2.5 mm—24.92	**<0.001**

**Table 16:** *p*-values for main effects obtained from the ANOVA analysis for tensile strength σ_M._

Effect	*p*-Value
Polymer type	0.141
Reinforcement	**<0.001**
Polymer content	**<0.001**

**Table 17:** *p*-values for main effects obtained from the Welch ANOVA analysis for maximum strain at failure ε_b._

Effect	*p*-Value
Polymer type	0.626
Reinforcement	**<0.001**
Polymer content	0.233

**Table 18:** Specific longitudinal elastic modulus *E_spec_* and specific strength σ*_spec_* of base materials, CFRTP composites, and TFMLs.

Material	*E_spec_*, GPa/(g/cm^3^)	σ*_spec_*, MPa/(g/cm^3^)
AA	27.53	119.99
PA6	0.68	27.06
PBT	1.95	35.02
PA6/1/10	1.88	49.02
PA6/0/10	7.96	73.84
A/PA6/35	17.73	90.03
A/PA6/1/35	21.34	91.42
A/PA6/0/35	22.27	106.46
PA6/1/05	1.96	65.67
PA6/0/05	7.89	115.30
A/PA6/25	23.51	95.45
A/PA6/1/25	22.15	105.14
A/PA6/0/25	23.88	131.96
PBT/1/10	2.62	50.13
PBT/0/10	6.51	80.33
A/PBT/35	17.11	71.28
A/PBT/1/35	19.65	75.48
A/PBT/0/35	18.07	85.06
PBT/1/05	2.68	57.99
PBT/0/05	7.03	102.48
A/PBT/25	16.31	73.06
A/PBT/1/25	18.12	92.06
A/PBT/0/25	21.43	124.08

**Table 19:** Comparison of experimental and model-predicted elastic modulus values with APE.

Material	E (exp), GPa	E (Model), GPa	APE, %
A/PA6/0/35	41.00	44.07	7.49
A/PA6/0/25	48.50	48.83	0.68
A/PA6/1/35	39.30	33.42	14.96
A/PA6/1/25	45.00	46.55	3.44
A/PBT/0/35	36.00	34.12	5.22
A/PBT/0/25	46.50	46.02	1.03
A/PBT/1/35	39.10	36.67	6.21
A/PBT/1/25	39.30	48.83	24.25

**Table 20:** Description of the fracture surfaces of woven thermoplastic composites.

Material	Fracture Surface Characteristics
PA6/0/10	– visible fiber bundles pulled out from the matrix, – lack of matrix residues on many fibers, – brittle-type matrix cracking, – broken and fragmented fibers of varying lengths, – locally porous regions with the presence of voids
PA6/0/05	– locally irregular fracture surface due to fiber bundles pulled out along the 0° direction, – brittle nature of the fracture, – fiber pull-out from the matrix, locally with residual matrix attached, – matrix cracking along the interfacial surface
PA6/1/10	– irregular fracture surface with local variations in height, – fibers pulled out from the matrix, devoid of polymer on their surface, – broken fibers, – local voids and micropores along the fracture zone
PA6/1/05	– irregular fracture surface with large height variations, – inclined cracks along the fabric structure, – absence of matrix between fiber bundles, – local areas with limited matrix coverage on the fibers, – -broken fibers, – brittle-type matrix cracking
PBT/0/10	– visible fiber bundles pulled out from the matrix, – lack of matrix residues on many fibers, – relatively flat fracture surface, – separation between fabric-rich and matrix-rich regions, – brittle-type matrix cracking
PBT/0/05	– locally irregular fracture surface due to fiber bundles pulled out along the 0° direction, – fiber pull-out, – separation between fabric-rich and matrix-rich regions, – brittle nature of the fracture
PBT/1/10	– irregular fracture surface, – fiber pull-out, – broken fibers, – voids between fiber bundles, – brittle-type matrix cracking
PBT/1/05	– irregular fracture surface with large height variations, – local delamination, – brittle nature of matrix cracking, – broken fibers of varying lengths, – fiber pull-outs, – detachment of individual fibers and entire fiber bundles

**Table 21:** Description of the fracture surfaces of TFMLs.

Material	Fracture Surface Characteristics
A/PA6	– delamination – the fracture surfaces of the outer aluminum layers are relatively smooth and flat, while the middle layer exhibits irregular depressions, – in the middle layer, “dimple fracture” features are visible; the outer layers also show signs of ductile failure, though to a lesser extent – the polymer layer exhibits a mixed fracture character, locally rough and in some areas smooth
A/PA6/0/35	– local delamination – individual air voids – fiber pull-out – exposed fiber surfaces – absence of matrix residues on selected fibers – broken fibers of irregular lengths – the upper and lower aluminum layers exhibit smooth fracture surfaces with slight local variations, while the middle layer is characterized by the most uniform and flat surface
A/PA6/0/25	– orderly fracture pattern with a clearly defined path along the metal–composite interface plane – dominant crack propagation along a single, relatively flat plane – no abrupt changes in fracture height – fiber pull-out – marks of pulled-out fiber bundles visible in the matrix – broken fibers – delamination at the interface between the adhesive film and the composite – the upper aluminum layer exhibits an irregular fracture with local protrusions, the middle layer is flat and relatively uniform, while the lower layer shows a rough surface with numerous indentations
,A/PA6/1/35	– delamination – numerous air bubbles – detachment of fiber bundles from the polymer matrix – broken fibers – the upper aluminum layer shows an irregular fracture with jagged fragments, the middle layer exhibits a smooth and uniform surface, while the lower layer, at 500× magnification, displays a rough structure with “dimple fracture” features
A/PA6/1/25	– delamination – matrix cracking (at the site of an air bubble) – irregular edge of the specimen – the fracture of the upper aluminum layer is irregular and jagged, the middle layer shows a porous structure with voids, and the lower layer has a rough surface with “dimple fracture” indentations – fiber pull-out – local areas with fibers devoid of matrix – marks of pulled-out fiber bundles visible in the matrix – broken fibers
A/PBT/25	– delamination – exposed metal areas devoid of polymer – irregular fracture pattern – the upper aluminum layer exhibits an irregular fracture with torn edge features, the middle layer is relatively smooth with local unevenness, and the lower layer has a continuous, elongated surface without distinct damage
A/PBT/0/35	– delamination between the aluminum layers and the composite layers – exposed carbon fibers devoid of polymer – broken fibers – fiber pull-out from the matrix – cracking of the polymer matrix in the composite – the upper aluminum layer shows an irregular fracture with “torn edge” features, the middle layer exhibits a relatively smooth fracture surface, while the lower layer displays a rough fracture with indentations characteristic of “dimple fracture”
A/PBT/0/25	– delamination – presence of loose carbon fiber bundles – broken fibers – fiber pull-out from the matrix – locally exposed carbon fibers devoid of polymer (some fibers are embedded in the matrix, while others are clearly pulled out) – broken fibers of varying lengths – the upper, middle, and lower aluminum layers exhibit relatively smooth fractures without noticeable jagged edges
A/PBT/1/35	– torn and heavily deformed adhesive film – delaminations occurring both at the adhesive–composite interface and at the adhesive–metal boundary – sharp and irregular polymer fracture edges – the fracture exhibits an asymmetric character – the upper, middle, and lower aluminum layers display a rough, porous fracture structure with an irregular pattern
A/PBT/1/25	– torn and heavily deformed adhesive film – delaminations occurring both at the adhesive–composite interface and at the adhesive–metal boundary – lack of visible polymer matrix residues on the fibers – the upper, middle, and lower aluminum layers exhibit relatively smooth and flat fracture surfaces

## Data Availability

Data are available from the corresponding author upon reasonable request.

## Abbreviations

The following abbreviations are used in this manuscript:

AA6061	Aluminum Alloy 6061
ANOVA	Analysis of Variance
CFRTP	Carbon Fiber-Reinforced Thermoplastic Composite
FEP	Fluorinated Ethylene–Propylene
FML	Fiber Metal Laminate
PA6	Polyamide 6
PA66	Polyamide 66
PBT	Polybutylene Terephthalate
SEI	Secondary Electron Imaging
SEM	Scanning Electron Microscopy
TFML	Thermoplastic Fiber Metal Laminate
UV	UV light
